# Re-Purposing the Ordering of Routine Laboratory Tests in Hospitalized Medical Patients (RePORT): protocol for a multicenter stepped-wedge cluster randomised trial to evaluate the impact of a multicomponent intervention bundle to reduce laboratory test over-utilization

**DOI:** 10.1186/s13012-024-01376-6

**Published:** 2024-07-02

**Authors:** Anshula Ambasta, Jayna M. Holroyd-Leduc, Surakshya Pokharel, Pamela Mathura, Andrew Wei-Yeh Shih, Henry T. Stelfox, Irene Ma, Mark Harrison, Braden Manns, Peter Faris, Tyler Williamson, Caley Shukalek, Maria Santana, Onyebuchi Omodon, Deirdre McCaughey, Narmin Kassam, Chris Naugler

**Affiliations:** 1 Department of Medicine, Cumming School of Medicine, University of Calgary, 3330 Hospital Dr NW, Calgary, AB T2N 4N1 Canada; 2 Department of Anesthesia, Pharmacology and Therapeutics, Therapeutics Initiative, University of British Columbia, Vancouver, V6T 1Z4 Canada; 3 Ward of the 21st Century, University of Calgary, GD01, CWPH,Building 3280 Hospital Drive NW, Calgary, AB T2N 4Z6 Canada; 4 Department of Medicine, University of Alberta, 116 St & 85 Ave, Edmonton, AB T6G 2R3 Canada; 5 Department of Pathology and Laboratory Medicine, University of British Columbia, Vancouver, V6T 1Z4 Canada; 6 Faculty of Medicine and Dentistry, University of Alberta, 116 St & 85 Ave, Edmonton, AB T6G 2R3 Canada; 7 Faculty of Pharmaceutical Sciences, University of British Columbia, Vancouver, V6T 1Z4 Canada; 8 Department of Community Health Sciences, Cumming School of Medicine, University of Calgary, 3330 Hospital Dr NW, Calgary, AB T2N 4N1 Canada; 9 Department of Pediatrics, Cumming School of Medicine, University of Calgary, 3330 Hospital Dr NW, Calgary, AB T2N 4N1 Canada; 10 Department of Pathology and Laboratory Medicine, Cumming School of Medicine, University of Calgary, 3330 Hospital Dr NW, Calgary, AB T2N 4N1 Canada

**Keywords:** Health care waste, Low value testing, Routine laboratory testing, Social comparison, Audit and Feedback, Implementation, Patient engagement

## Abstract

**Background:**

Laboratory test overuse in hospitals is a form of healthcare waste that also harms patients. Developing and evaluating interventions to reduce this form of healthcare waste is critical. We detail the protocol for our study which aims to implement and evaluate the impact of an evidence-based, multicomponent intervention bundle on repetitive use of routine laboratory testing in hospitalized medical patients across adult hospitals in the province of British Columbia, Canada.

**Methods:**

We have designed a stepped-wedge cluster randomized trial to assess the impact of a multicomponent intervention bundle across 16 hospitals in the province of British Columbia in Canada. We will use the Knowledge to Action cycle to guide implementation and the RE-AIM framework to guide evaluation of the intervention bundle. The primary outcome will be the number of routine laboratory tests ordered per patient-day in the intervention versus control periods. Secondary outcome measures will assess implementation fidelity, number of all common laboratory tests used, impact on healthcare costs, and safety outcomes. The study will include patients admitted to adult medical wards (internal medicine or family medicine) and healthcare providers working in these wards within the participating hospitals. After a baseline period of 24 weeks, we will conduct a 16-week pilot at one hospital site. A new cluster (containing approximately 2–3 hospitals) will receive the intervention every 12 weeks. We will evaluate the sustainability of implementation at 24 weeks post implementation of the final cluster. Using intention to treat, we will use generalized linear mixed models for analysis to evaluate the impact of the intervention on outcomes.

**Discussion:**

The study builds upon a multicomponent intervention bundle that has previously demonstrated effectiveness. The elements of the intervention bundle are easily adaptable to other settings, facilitating future adoption in wider contexts. The study outputs are expected to have a positive impact as they will reduce usage of repetitive laboratory tests and provide empirically supported measures and tools for accomplishing this work.

**Trial Registration:**

This study was prospectively registered on April 8, 2024, via ClinicalTrials.gov Protocols Registration and Results System (NCT06359587). https://classic.clinicaltrials.gov/ct2/show/NCT06359587?term=NCT06359587&recrs=ab&draw=2&rank=1

**Supplementary Information:**

The online version contains supplementary material available at 10.1186/s13012-024-01376-6.

Contributions to the literature • The RePORT study leverages current evidence-based intervention tools and builds on those to implement and evaluate their effect in a real-world implementation study

• Limited studies have comprehensively evaluated the effect (including potential unintended consequences) of intervening on laboratory test use in hospitals

• With the growing problem of overuse in healthcare systems, this study offers a model of how implementation strategies can be applied and evaluated for de-implementation projects

• Our study incorporates patient engagement through all phases of the research, showcasing a way of integrating authentic patient engagement with implementation science

## Background

Laboratory tests are an indispensable tool in medicine for diagnosing and monitoring clinical conditions [1]. Yet, between 16–56% of laboratory-based testing is deemed wasteful as it does not advance patient care [1]. One form of laboratory test over-use is the use of repetitive routine blood testing in hospitalized patients [2, 3]. In addition to contributing to healthcare waste and a downstream cascade of more tests and procedures, laboratory test over-utilization in hospitals is associated with patient discomfort, disruption in sleep patterns, and hospital-acquired anemia which can increase the risk of transfusions, prolong length of stay, and increase mortality [4–10]. Reducing laboratory test over-utilization in hospitals thus provides an avenue towards reducing health care waste without compromising patient care [11].

Choosing Wisely Canada, a campaign to reduce unnecessary tests and treatments in healthcare has identified repetitive laboratory testing in hospitalized patients as low-value care and made several recommendations to reduce laboratory test over-use [12–14]. These recommendations need active and comprehensive de-implementation efforts to facilitate sustainable change [15–19]. Many described interventions have been shown to reduce laboratory test over-utilization, with the greatest evidence for multicomponent initiatives [20–27]. However, most studies are small and there is little systematic evaluation of downstream consequences and sustainability. A recent meta-analysis of interventions to reduce laboratory test over-utilization indicates that interventions such as education, cost display, and policy change were effective in multi-faceted intervention settings [28]. In terms of effectiveness and sustainability, changes to electronic medical record (EMR) systems were deemed as the most valuable single intervention [28].

Our group has iteratively developed a multicomponent intervention bundle (including healthcare provider and patient engagement strategies) to safely reduce repetitive use of routine laboratory testing in hospitals. Earlier versions of this intervention bundle have demonstrated safe reduction in routine laboratory test utilization in a pilot study [29], followed by expansion across eight units and four tertiary care hospitals [30]. A definitive large-scale implementation study is needed to determine the impact and outcomes of this intervention bundle. Our Canadian Institutes of Health Research (CIHR) funded project, Re-Purposing the Ordering of Routine laboratory Tests (RePORT) evaluates the impact of an enhanced and innovative intervention bundle in a wider context and adapted to local hospital context, in a multicenter clinical trial using a stepped-wedge cluster randomized design with number of routine laboratory tests per patient-day as the primary outcome. We provide below an overview of the intervention bundle tools, our implementation strategy and associated implementation science frameworks, and detail the study design and planned analysis for this implementation protocol.

## Methods/Design

### Study setting and population

We report this protocol using the SPIRIT guidelines [31] for reporting of intervention trials (see Additional file 1). This study will be conducted in the adult medical wards of 16 hospitals representing academic practice and community settings across the province of British Columbia (BC) in Canada (Table 1). The BC Ministry of Health works together with five regional health authorities [32] to deliver acute care in hospitals through a publicly funded system. The 16 hospitals represent all five health authorities within BC. The study population will include all healthcare providers and patients hospitalized in adult medical wards. Medical wards include patients admitted under Internal Medicine and Family Medicine (Hospitalist) groups and exclude patients admitted to Critical Care, Surgical, Pediatric, Obstetric, and specialized tertiary care medical sub-specialty units. These patients are excluded owing to their unique requirements in specialized units.

**Table 1:** List of participating facilities and characteristics in the province of British Columbia, Canada

Facility Name	Website	City	Operator Type and Identity^a^	Hospital Type^b^	Academic Affiliation (i.e. Has Teaching units)	Number of Hospital Beds
Vancouver General Hospital	https://www.vch.ca/en/location/vancouver-general-hospital	Vancouver	VCHRI/VCHA	General	Yes	666
Lions Gate Hospital	https://www.vch.ca/en/location/lions-gate-hospital	North Vancouver	VCHRI/VCHA	General	No	268
Richmond Hospital	Hospital Will go live in Summer 2024	Richmond	VCHRI/VCHA	General	No	220
University of British Columbia Hospital	https://www.vch.ca/en/location/ubc-hospital	Vancouver	VCHRI/VCHA	General and Auxillary	Yes	87
Mount Saint Joseph Hospital	https://www.providencehealthcare.org/hospitals-residences/mount-saint-joseph-hospital	Vancouver	Providence Health Care	Auxillary	No	101
St. Paul’s Hospital	https://www.providencehealthcare.org/hospitals-residences/st-paul%27s-hospital	Vancouver	Providence Health Care	General	Yes	451
University Hospital of Northern British Columbia (UHNBC)	https://www.northernhealth.ca/find-a-facility/hospitals/university-hospital-northern-british-columbia-uhnbc	Prince George	Northern Health	General	Yes	219?
Royal Jubilee Hospital	https://www.islandhealth.ca/our-locations/hospitals-health-centre-locations/royal-jubilee-hospital-rjh	Victoria	Island Health	General	No	500
Victoria General Hospital	https://www.islandhealth.ca/our-locations/hospitals-health-centre-locations/victoria-general-hospital-vgh	Victoria	Island Health	General	No	414 (344 acute care beds, 30 Neuro-Rehabilitation beds and 40 Geriatric Ward beds)
Royal Inland Hospital (Kamloops)	https://www.interiorhealth.ca/locations/royal-inland-hospital	Kamloops	Interior Health	General	Yes	254
Kelowna General Hospital	https://www.interiorhealth.ca/locations/kelowna-general-hospital	Kelowna	Interior Health	General	No	526
Abbotsford Regional Hospital and Cancer Centre	https://www.fraserhealth.ca/Service-Directory/Locations/Abbotsford/abbotsford-regional-hospital-and-cancer-centre	Abbotsford	Fraser Health	General	Yes	300
Chilliwack General Hospital	https://www.fraserhealth.ca/Service-Directory/Locations/Chilliwack/chilliwack-general-hospital	Chilliwack	Fraser Health	General and Auxiliary	Yes	336
Peace Arch Hospital	https://www.fraserhealth.ca/Service-Directory/Locations/White-Rock/peace-arch-hospital	White Rock	Fraser Health	General	Yes	146
Royal Columbian Hospital	https://www.fraserhealth.ca/Service-Directory/Locations/New-Westminster/royal-columbian-hospital	New Westminster	Fraser Health	General	Yes	402
Surrey Memorial Hospital	https://www.fraserhealth.ca/Service-Directory/Locations/Surrey/surrey-memorial-hospital	Surrey	Fraser Health	General	Yes	650

^a^Operator Type and Identity Options: Vancouver Coastal Health (VCHRI/VCHA)

^b^Hospital Type Options: General = Active Treatment, Auxiliary = Long Term Care/Chronic Care)

### Intervention bundle evolution

We have identified six target routine tests (complete blood count, electrolytes, creatinine, urea, international normalized ratio, and partial thromboplastin time) that collectively constitute 40% of expenditure on hospital-based laboratory tests in medical units [33]. In our previously published pilot study [29], we had used local consensus to develop appropriate use criteria for these target tests and developed an online case-based module and pocket cards summarizing these recommendations. The educational module and pocket-cards were coupled with electronically distributed laboratory test utilization data for individual physicians and aggregate learner (medical students and resident physicians) teams to form the intervention bundle.

After the pilot, we developed national expert consensus-based recommendations on appropriate use of the target tests [33], and used these recommendations to create a revised interactive, online case-based educational module [34] that is accredited by the Royal College of Physicians and Surgeons of Canada for continuing professional development credits. Our intervention bundle during our subsequent expansion across eight units [30] included the case-based educational module. The electronically distributed laboratory test utilization data for physicians and learners was enhanced with the opportunity for physicians to join virtual facilitated group audit and group feedback sessions conducted according to the Calgary Audit and Feedback framework [35, 36].

Our current multimodal intervention bundle builds on the prior educational and audit and feedback tools and adds in tools to engage patients and harness Electronic Medical Record (EMR) ordering processes.

### Intervention bundle components

Our multicomponent intervention bundle (please see Additional File 2 for details) includes the following components:

(i) Education: We will use an updated online case-based module (Additional File 2) that builds on our learnings from our prior studies and continues to maintain accreditation through the Royal College of Physicians and Surgeons of Canada for continuing professional development credits. The online module will be paired with a framework for purposeful utilization of laboratory tests to create a clinical decision support tool (Appendix File 2) [37].
(ii) Audit and feedback: We will also provide laboratory test utilization data to healthcare providers. These data reports will be at the individual, group, or ward-level based on site preference and feasibility. We have designed facilitated sessions to review the data in a group setting according to the Calgary Audit and Feedback Framework [35, 36]. During these one-hour long sessions, facilitators will lead participants through different phases of reacting to data, seeking to understand, question, justify, and contextualize, and then finally moving to reflection and planning for change. This is an evidence-informed approach for implementing learning interventions using Audit and Feedback, with demonstrated efficacy [38, 39]. These sessions incorporate specific behaviour change techniques in Audit and Feedback that target behaviour change [40].
(iii) Electronic Medical Record (EMR) ordering process changes: Our partnerships with clinical informatics leaders in BC will enable our team to review EMR ordering processes, provide education to healthcare providers on how to effectively harness EMR systems to purposefully order routine laboratory tests, and facilitate dialogue and change around suggestions from healthcare providers on EMR ordering systems.
(iv) Patient engagement: Our research team has collaborated with patient research partners through a Patient Advisory Council to create tools to engage patients with laboratory testing in hospitals. These tools include a patient infographic [41] (Additional file 2), an educational video [41], and a website [42] (www.hospitalbloodwork.ca) that collectively aim to educate patients about the process of laboratory testing in hospitals, and ways that they can seek engagement with the process. We operationalized the Patient Engagement Framework from Canadian Institute of Health Research’s Strategy for Patient Oriented Research [43], attending to the principles of patient engagement around inclusiveness, support, mutual respect, and co-build [44]. Using the validated Patient and Public Engagement evaluation tool [45], we have also conducted a formal evaluation of patient engagement within our council to foster ongoing engagement [46]. The evaluation indicated that patient research partners felt heard and engaged with the work of the council [46].

Patient research partners were co-applicants in the initial proposal seeking funding for this work, co-conducted with the research team a qualitative study to understand patient perceptions of hospital-based laboratory testing [47], and co-designed patient engagement tools with a human-centered design expert based on the findings from the qualitative study. Patient research partners have taken the lead in disseminating the results of their work through presentations at international (Preventing Overdiagnosis Conference, Denmark 2023 [48]) and national conferences (Northwest Strategy for Patient Oriented Research Collaborative Forum in Edmonton 2023 [49] and Putting Patients First Conference in Vancouver 2023 [50]). Patient research partners will continue to work alongside the research team with implementation and evaluation of patient engagement tools.

### Study design

This multicenter, quasi-experimental stepped-wedge cluster randomized trial [51] will assess whether a multicomponent intervention bundle can reduce the number of target routine laboratory tests ordered in hospitalized medical patients. Implementation of the intervention bundle will be guided by the Knowledge to Action cycle [52], where we will understand the local context, assess local barriers and facilitators, and select and tailor our intervention tools. We will use the RE-AIM (Reach, Effectiveness, Adoption, Implementation and Maintenance) framework [53, 54] to evaluate the intervention bundle and the National Health Service (NHS) Sustainability model to plan for sustainment [55].

**Fig. 1:**
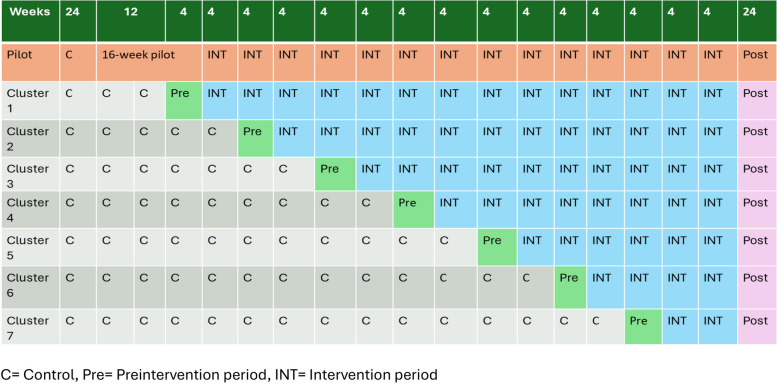
Stepped-wedge cluster randomized trial study design for the intervention bundle. C= Control, Pre= Preintervention period, INT= Intervention period

The study will first conduct a feasibility assessment of the intervention bundle tools over a 16-week pilot period at one site. The pilot period will provide an opportunity for testing the intervention tools and developing implementation strategies. After the pilot, each cluster will enter the implementation stage in 12-week steps (Fig. 1) in a random order determined by computerized random number generation. Each cluster will contain approximately 2 hospitals, loosely grouped based on geographical proximity. A cluster will be eligible to enter randomization for implementation once local data infrastructure is complete. The initial four weeks of implementation will serve as the pre-intervention period, which will be followed by an 8-week intervention period. The final four weeks of the intervention period for one cluster will overlap with the pre-intervention period of the following cluster (Fig. 1).

**Fig. 2:**
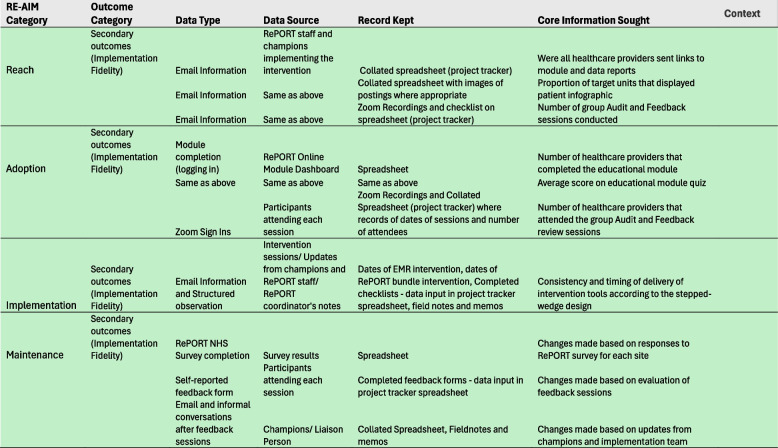
Implementation Process Evaluation Tool

During the pre-intervention period, we will finalize multidisciplinary implementation teams including nurse managers and educators, ward directors, physicians, resident physicians, and medical students at each site. Local implementation teams will provide information on local barriers and facilitators which will allow for selection and tailoring of intervention tools. They will receive virtual implementation support by the research team and the Patient Advisory Council to adapt and deliver intervention bundle tools within local context. Different intervention tools may be used at each site depending on local context and adapted to local barriers and facilitators. Each site will receive at least one tool for healthcare provider engagement and one for patient engagement. All site-specific modifications of intervention tools will be noted in our implementation process tracking tool (Fig. 2). Members of implementation teams will also complete a combined survey to assess readiness for implementation (based on the Ready, Set, Change! Decision support tool [56]) and determine overall climate of sustainability and strategies to enable sustainability of the intervention (based on NHS Sustainability survey [57]) (Additional file 3).

Following pre-intervention, clusters will enter an 8-week intervention period where intervention tools will be deployed. Implementation of intervention bundle tools will be integrated with existing hospital processes such as ongoing faculty rounds, business meetings, site and ward meetings, quality councils and existing communication forums. Local study champions (members of the implementation teams) will help disseminate educational materials including link to the online module, clinical decision support tool and patient engagement tools. Study brochures will be shared electronically, and patient infographic with QR code linking to project website will be displayed in physical hospital wards. Each healthcare provider will receive an email with links to online project educational resources. Lab utilization reports (at individual/provider group/ward level) will be sent at a frequency ranging between every 3–6 months, decided by each site. Virtual facilitated sessions will be organized to discuss the data according to the Calgary Audit and Feedback framework [35, 36].

Following implementation completion in the final cluster, we will collect 24 weeks of post-implementation data. This phase aims to re-evaluate impact of integrated implementation tools in the absence of dedicated personnel support at the end of 24 weeks post-implementation. Access to educational resources will be continued for healthcare providers and patients through embedded processes such as divisional websites, onboarding packages for new faculty and resident physicians, and physical posters with QR codes in workspaces. Laboratory test utilization data reports will be sent with links to educational resources at a frequency chosen by each site.

The stepped wedge design allows for robust evaluation of the intervention bundle while factoring in the logistical constraints common to implementation studies [58]. This study permits rigorous evaluation of the intervention bundle while enabling all willing hospital facilities to receive the intervention. Enrollment will occur at the hospital level. We do not anticipate any attrition in study sites. We have operational approval and support from regional health authority leadership and local study champions. In the event that a site does drop out for a logistical reason, we have adequate numbers of other sites with similar characteristics that could be recruited. The nature of the intervention makes it impossible to mask research team members and sites to the assignment of intervention when implementation begins.

### Outcomes and data sources

We will use the RE-AIM framework to guide our evaluation of the intervention bundle (see Table 2). This framework offers a systematic approach to the collection and analysis of data required to plan, implement, evaluate, and strategize sustainability of the intervention. The primary outcome will be the number of target routine laboratory test orders per patient-day with implementation of the intervention bundle compared to the control period. Secondary outcomes will assess number of all common laboratory tests (number of tests that collectively contribute to more than 80% of test utilization in hospitals), costs associated with target routine laboratory testing, implementation fidelity and potential unintended consequences of the intervention (Table 2). We will use our implementation process tracking tool to track fidelity of implementation and implementation context (Fig. 2). We will use administrative data for quantitative outcomes and complement that with data obtained from surveys (readiness assessment and sustainability survey), observational field notes and memos collected by study coordinator through our implementation process tool, and qualitative data collected during our facilitated utilization data review sessions. An evaluation of all outcomes listed in Table 2 will be conducted after study implementation is complete and the primary outcome will be re-evaluated at 24 weeks after study completion to assess for sustainability.

**Table 2:** Outcomes, potential unintended consequences, and implementation fidelity

RE-AIM Category	Outcome category	Outcome definition
Effectiveness	Primary outcome	• Number of target routine laboratory tests drawn per patient-day
Effectiveness	Secondary outcome	• Number of all common laboratory tests (tests that collectively comprise > 80% of laboratory test utilization in hospitals) drawn per patient-day
		• Costs associated with routine laboratory testing
Effectiveness	Secondary outcomes (Potential unintended consequences)	• In-hospital mortality
		• 30-day post discharge mortality
		• 30-day readmission
Reach	Secondary outcomes (Implementation Fidelity)	• Proportion of healthcare providers that were sent links to module and data reports
		• Proportion of target units that displayed patient infographic
		• Number of group Audit and Feedback sessions conducted
Adoption		• Number of healthcare providers that completed the educational module
		• Average score on educational module quiz
		• Number of healthcare providers that attended the group Audit and Feedback review sessions
Implementation		• Consistency and timing of delivery of intervention tools according to the stepped-wedge design
Maintenance		• Changed made based on responses to surveys for each site, responses to evaluation forms at each audit and feedback session, and conversations with champions/implementation team

### Sample size calculation

Based on administrative data, we estimate a mean of three target laboratory test orders per patient at baseline, and an average of 100 patients per site per day on the medical wards. We believe that a 10% reduction in mean number of target test orders per patient-day is clinically important. With 16 participating sites, 8-week intervention periods, and an intra-cluster correlation of 0.1 based on our prior work or even as high as 0.2 to account for geographical clustering, we will have the ability to detect a difference of 10% in our primary outcome with greater than 90% power (2-sided alpha of 0.05) [59].

### Statistical data analysis

We will use generalized linear mixed models throughout the analysis to account for multiple observations per patient and shared characteristics (correlation) between geographical clusters [60]. Models will be adjusted for patient age, sex, case-mix group [61], number of patient-days and hospital type. Using intention to treat, clusters will be analyzed according to their randomized cross over time from control to intervention period, with preintervention period data being excluded as washout. Calendar time will be included as a fixed effect for each step to account for secular trends. We anticipate using logistic regression (via a logit link) for binary outcomes (inpatient mortality, 30-day readmission, 30-day mortality), Poisson regression for count variables (e.g. number of tests) and linear regression models for continuous outcomes (e.g. length of stay). A p-value < 0.05 will be considered statistically significant. We will use complementary data from our surveys, data and field notes collected through the implementation process tracking tool, and qualitative data collected during the facilitated utilization data review sessions to help interpret site-specific results.

### Costing analysis

We will compare costs of care and patient outcomes with and without implementation of the intervention bundle from the perspective of the publicly funded healthcare system. We will include costs of: (i) target laboratory tests with and without implementation of the intervention bundle using a cost per test or test group obtained from published reference median costs [62]; (ii) the index hospitalization (hospitalization where the implementation occurs) for each patient using micro-costing data [63] to provide a detailed cost per patient, including all resources consumed during the hospital stay such as direct and indirect nursing costs, surgery, ancillary services (physiotherapy, occupational therapy), and laboratory and drug costs; (iii) post-hospitalization healthcare costs over the 30-day period following discharge from hospital for the index hospitalization including: subsequent hospitalizations, emergency departments visits, ambulatory care, and urgent care costs. This will be done to estimate unintended downstream consequences of intervening on laboratory test use. We will present costs by category, and the differences in total costs will be calculated and compared to implementation costs. Our analysis will be a detailed cost consequence analysis, presented in conjunction with other outcomes.

## Discussion

As healthcare costs increase globally, we need context-specific de-implementation strategies to reduce low-value healthcare practices and integrate effective, evidence-based approaches. Our study is one of few implementation science studies that combines real-world operational change with a rigorous scientific evaluation approach to comprehensively study the de-implementation of the practice of repetitive use of routine laboratory testing in hospitals. Specifically, the study builds on intervention tools that have previously demonstrated success with reduction of redundant laboratory testing in hospitals [29, 30], and capitalizes on the momentum created by the current national campaign headed by Choosing Wisely Canada to ‘*Use Labs Wisely’ * [64].

While numerous studies have focused on reducing repetitive laboratory testing in hospitals, most are small, single institution efforts without clear plan for sustainability or scale [28, 65]. Our study is a robust effort to build upon prior work in a systematic manner that allows for scale and plan for sustainability. A recent systematic review on interventions to reduce repetitive ordering of low-value inpatient laboratory tests identified EMR and policy changes as the most effective and sustainable [28]. However, providers found the use of strict limitations and unavoidable pop-up alerts unfavourable. Moreover, focused EMR or policy changes are easier for laboratory tests that have clearly defined appropriateness criteria or re-testing intervals.. In contrast, the tests we focus on for our study are common tests with wide applicability, it is only their redundant use that is low-value [37, 66]. As such, our multicomponent intervention bundle which combines education, audit and feedback, EMR changes and patient engagement is novel in the area of repetitive laboratory test utilization and will contribute to our understanding of how the different elements can interact and contribute to reducing repetitive laboratory test utilization, while engaging and winning the favour of practising providers. Our study will be one of the few that has developed de-novo patient engagement tools in this field and will report on the evaluation of those tools as part of a multicomponent intervention bundle. In an era of increasing patient access to laboratory test results, patient engagement tools to empower and engage patients with appropriate use is timely.

While audit and feedback is a widely used strategy to improve professional practice and has been used as a component of other multimodal quality improvement studies, there exists considerable heterogeneity in the methodologies used [67]. The effectiveness of audit and feedback depends on baseline performance and method of delivery of the feedback [67]. In our study, we use an established framework of delivering audit and feedback (Calgary Audit and Feedback Framework) which has demonstrated prior efficacy [30, 38, 39]. This study will contribute towards building evidence around a specific method of delivering audit and feedback which can be reliably replicated and adapted based on evolving evidence. Hence our study will help advance the science of delivery of audit and feedback. In addition, our use of virtual facilitation for the group audit and feedback sessions to enable province-wide implementation will contribute towards evidence in the area of virtual application of facilitated audit and group feedback based on the Calgary Audit and Feedback Framework. A recent scoping review on virtual facilitation identified significant variability on reporting of virtual facilitation and lack of details on how it was conducted [68]. Our study will contribute towards literature in the development and evaluation of effective virtual facilitation to improve uptake of interventions, both through the application of virtual facilitation for implementation support, and for the facilitated audit and feedback sessions.

Our study is also one of few studies that includes patient engagement embedded in every aspect of the research and includes co-designed intervention tools that will be implemented. This trial will contribute to the understanding of how to apply the science of Knowledge Translation to de-implementation [69], including delivery of Audit and Feedback reports, design of patient engagement strategies, and evaluation of tailored pragmatic multicomponent strategies. To the best of our knowledge, this is one of the largest planned de-implementation trials focusing on reducing redundant use of routine daily laboratory testing in hospitals. Results of the study have the potential to improve the quality of care for hospitalized patients. Lessons learned will help inform future implementation of other interventions in hospitals in general, a treatment setting that deals with high-cost and high-acuity care [70]. Given the lack of evidence on why and how implementation strategies work, our complementary data to understand local contextual determinants will advance the science of implementation.

Results of our study will be disseminated to healthcare providers and researchers through presentations at national and international conferences and open-access publications in peer-reviewed journals. Furthermore, we plan to have a cross-sectoral meeting with interested policymakers, healthcare providers and patient participants after the end of the study to disseminate experiences and findings. We will also conduct a press release after publication to share information with the public. We will include project metadata in the supplementary appendix of published articles.

Given the stepped-wedge design, a notable limitation is potential confounding due to temporal trends as more clusters are exposed to the intervention with study progress [58]. To counter this, we will use randomization to determine the sequence of cluster entry into intervention. In this study, hospital is the unit of randomization, therefore contamination with physicians and medical trainees working in multiple hospitals is possible. The study will report on the effect of this intervention in a population of hospitalized adult medical patients in a single province in Canada, as such, results may not be generalizable to other patient populations and settings.

## Trial status

Pilot implementation since May 6th 2024.

### Supplementary Information

Supplementary Material 1. 

Supplementary Material 2. 

Supplementary Material 3. 

## Data Availability

The final trial data set will be accessed by the study PI (AA), project team statisticians and data analysts. All administrative data used in this study are available from the provincial and institutional health authority data analytics sources and can be requested in accordance with institutional policies and procedures. All other data (including qualitative data) are available from the corresponding author upon reasonable request.

## Abbreviations

RePORT: Repurposing the Ordering of Routine Laboratory Tests in Hospitalized Medical Patients
EMR: Electronic Medical Record
