# Supplementary material for: Re-Purposing the Ordering of Routine Laboratory Tests in Hospitalized Medical Patients (RePORT): protocol for a multicenter stepped-wedge cluster randomised trial to evaluate the impact of a multicomponent intervention bundle to reduce laboratory test over-utilization
Source: Implement Sci. 2024 Jul 2;19:45. doi: 10.1186/s13012-024-01376-6 (PMC11221016; doi:10.1186/s13012-024-01376-6)
Supplement: Supplementary file 2 — Supplementary Material 2. [file 13012_2024_1376_MOESM2_ESM.pdf]

Additional File 2: RePORT Study Intervention Bundle Components

Contents

1. Education
    a. Online Interactive Case-based Educational Module
    b. Clinical Decision Support Tool
2. Audit and Feedback
3. EMR Ordering Process Changes
4. Patient Engagement
    a. Patient Infographic
    b. Patient Video

1a. Online Interactive Case-based Educational Module:
https://cards.ucalgary.ca/deck/432

Cards (/)

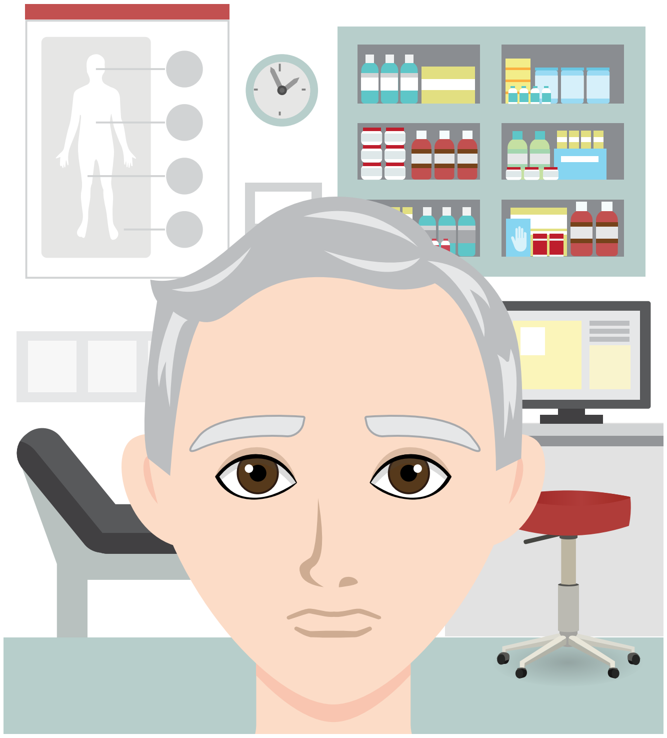

Welcome to the Self-Assessment Program on Optimization of laboratory test utilization amongst inpatients.

This module walks through 7 case examples followed by 7 skill-testing questions to teach key points on optimization of laboratory test utilization.

This activity is an Accredited Self-Assessment Program (Section 3) as defined by the Maintenance of Certification Program of the Royal College of Physicians and Surgeons of Canada. This module will take an hour to complete. When module completion is combined with in-person attendance at a facilitated feedback session, you may claim a maximum of 2 hours. To attend a facilitated feedback session email: aambasta@ucalgary.ca

Ready to begin?

Yes

Submit

All content © 2020 University of Calgary

Cards (/)

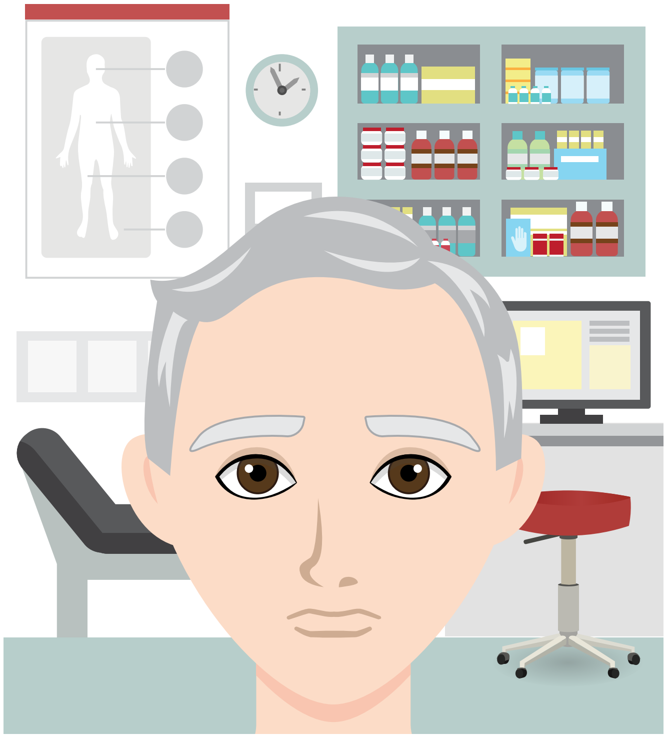

Welcome to the Self-Assessment Program on Optimization of laboratory test utilization amongst inpatients.

This module walks through 7 case examples followed by 7 skill-testing questions to teach key points on optimization of laboratory test utilization.

This activity is an Accredited Self-Assessment Program (Section 3) as defined by the Maintenance of Certification Program of the Royal College of Physicians and Surgeons of Canada. This module will take an hour to complete. When module completion is combined with in-person attendance at a facilitated feedback session, you may claim a maximum of 2 hours. To attend a facilitated feedback session email: aambasta@ucalgary.ca

Ready to begin?

Yes

Incorrect

Each *card* you play will be accompanied with some feedback. After you answer a *card* question, we will display your sequence in the deck below.

You can register using the link below, and starting this deck again, and once complete, you'll recieve a CME attendance certificate. This will also allow automatically save your progress if you wish to finish this module at a later time.

Alternatively, if you finish the deck in this open access mode, you'll have an opportunity to enter your name, email and institution so we can provide a CME attendance certificate.

This is card 1 of 23.

Click next to proceed.

Pick A Deck (/collection)   Next Card (/next/)

Register Now

Why not start keeping score? Registration is quick and easy.

(/register)

Authors

Dr. Anshula Ambasta

aambasta@ucalgary.ca (mailto:aambasta@ucalgary.ca)

Dr. Anita Palepu

apalepu@hivnet.ubc.ca (mailto:apalepu@hivnet.ubc.ca)

Dr. Cathy Code

ccode@toh.ca (mailto:ccode@toh.ca)

Dr. Todd Lee

Todd.lee@medsafer.org (mailto:Todd.lee@medsafer.org)

Dr. Liberty Liu

Liberty.liu@ahs.ca (mailto:Liberty.liu@ahs.ca)

Dr. Douglas Woodhouse

Douglas.woodhouse@ucalgary.ca (mailto:Douglas.woodhouse@ucalgary.ca)

Dr. Irene Ma

ima@ucalgary.ca (mailto:ima@ucalgary.ca)

UNIVERSITY OF CALGARY
CUMMING SCHOOL OF MEDICINE

All content © 2020 University of Calgary

Cards (/)

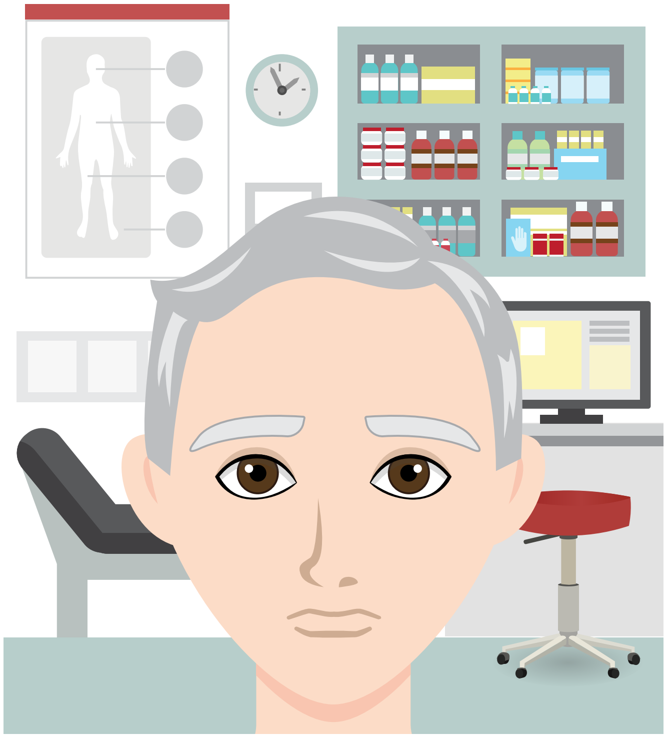

Mr. A is an 85 year old man who is admitted to hospital with a new diagnosis of pneumonia. Admission orders include a standing order for daily routine blood tests (CBC, electrolytes, creatinine, urea, INR and PTT) for the rest of his admission stay.

What are the potential downstream effects of repetitive routine blood tests in Mr. A? (choose all that apply)

Patient discomfort

Hospital acquired-anemia

Unnecessary transfusions

Prolonged hospitalization

Increased mortality

None of the above

Submit

UNIVERSITY OF CALGARY
CUMMING SCHOOL OF MEDICINE

All content © 2020 University of Calgary

Cards (/)

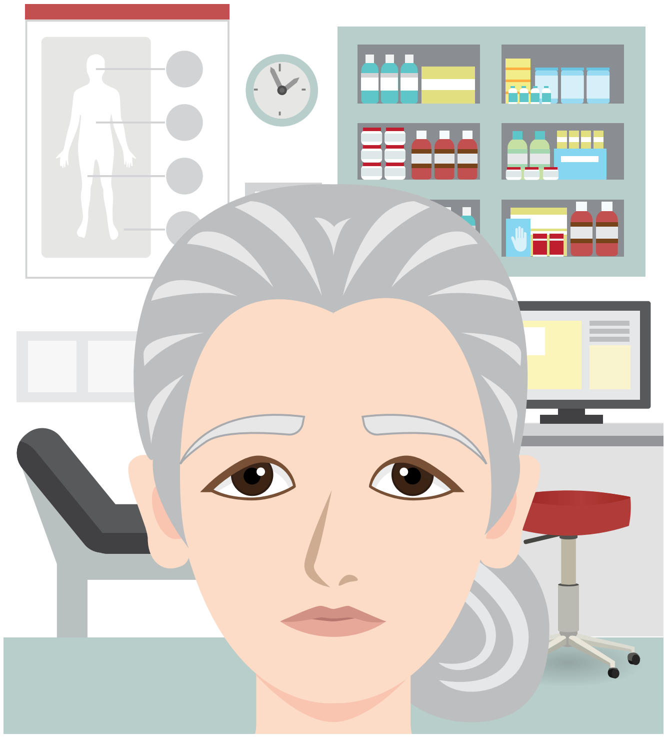

Ms. B is a 90 year old woman who presents with progressive dyspnea, orthopnea, and peripheral edema.

Past medical history:

Hypertension

Diabetes Mellitus type 2

Bilateral hip osteoarthritis

Gout

Current medications:

hydrochlorothiazide 25 mg daily, metformin 1000mg bid, gliclazide 80mg bid, allopurinol 300mg/day, acetaminophen prn

Examination: T37.1ºC, HR 105, RR 24, BP 170/70, O2 saturation 84% on room air with increased work of breathing. Elevated JVP, bilateral crackles on lung auscultation, and peripheral pitting edema to her knees.

Lab results:

Hemoglobin: 110 g/L (120-160)

Leukocytes: 12 x 10^9/L (3.5-12)

Platelets: 210 x 10^9/L (150-400)

Sodium: 138 mmol/L (135-145)

Potassium: 4.0 mmol/L (3.5-5.1)

Creatinine: 47 µmol/L (50-110)

GFR 115 mL/min/1.73m^2

Other tests:

Chest X-ray: pulmonary edema

She is admitted for heart failure and treated with an initial intravenous bolus dose of furosemide, followed by daily diuretic therapy

How often should you check her creatinine and electrolytes in hospital?

No further testing required

Daily for the next few days depending on results and diuresis plan

Every 12 hours for the next few days depending on results and diuresis plan

Daily for the rest of her hospitalization

Submit

UNIVERSITY OF CALGARY
CUMMING SCHOOL OF MEDICINE

All content © 2020 University of Calgary

Cards (/)

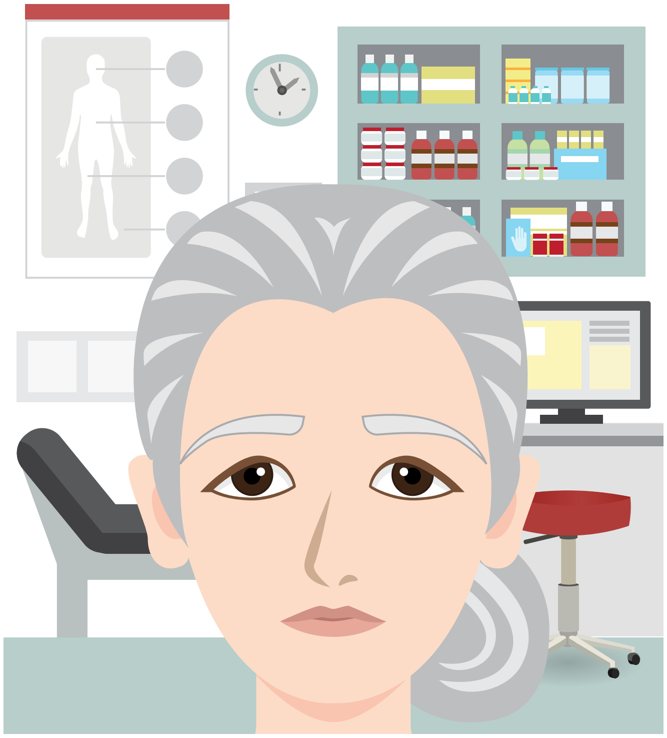

Ms. B is a 90 year old woman who presents with progressive dyspnea, orthopnea, and peripheral edema.

Past medical history:

Hypertension

Diabetes Mellitus type 2

Bilateral hip osteoarthritis

Gout

Current medications:

hydrochlorothiazide 25 mg daily, metformin 1000mg bid, gliclazide 80mg bid, allopurinol 300mg/day, acetaminophen prn

Examination: T37.1ºC, HR 105, RR 24, BP 170/70, O2 saturation 84% on room air with increased work of breathing. Elevated JVP, bilateral crackles on lung auscultation, and peripheral pitting edema to her knees.

Lab results:

Hemoglobin: 110 g/L (120-160)

Leukocytes: 12 x 10^9/L (3.5-12)

Platelets: 210 x 10^9/L (150-400)

Sodium: 138 mmol/L (135-145)

Potassium: 4.0 mmol/L (3.5-5.1)

Creatinine: 47 µmol/L (50-110)

GFR 115 mL/min/1.73m^2

Other tests:

Chest X-ray: pulmonary edema

She is admitted for heart failure and treated with an initial intravenous bolus dose of furosemide, followed by daily diuretic therapy

Update:

Ms. B is subsequently diagnosed with heart failure with preserved ejection fraction secondary to poorly controlled hypertension.

She is successfully diuresed to euvolemia and her blood pressure control is improved with the addition of amlodipine. The furosemide is stopped and she is transitioned back to her home medications.

Physiotherapy team advises you that Mrs. B will need an additional week of focused rehabilitation prior to discharge.

How often should Ms. B have bloodwork (CBC, electrolytes, creatinine) performed during this week in hospital?

Every day

Every 2 days

Every 3 days

At the beginning of the week and prior to discharge

None, unless indicated based on clinical assessment

Submit

UNIVERSITY OF CALGARY
CUMMING SCHOOL OF MEDICINE

All content © 2020 University of Calgary

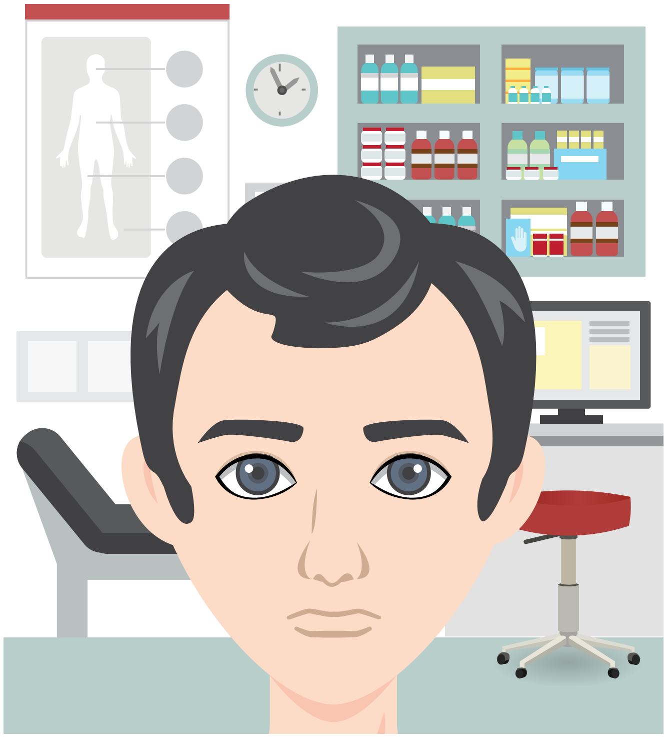

Mr. C is a 45 year old man with longstanding diabetes mellitus type 1 who presents to hospital with abdominal pain, vomiting, and elevated blood glucose readings at home.

Past medical history:

End-stage kidney disease secondary to diabetes, on 3X/week hemodialysis, no residual renal function

Peripheral Neuropathy

Hypertension

Dyslipidemia

Current medications: gabapentin 100mg tid, amlodipine 10mg daily, hydralazine 25 mg tid, rosuvastatin 5mg daily, polysaccharide iron complex 150 mg daily, darbepoetin alfa 30mcg/week

Examination: T36.5ºC, HR 130, RR 32, BP 100/64, O2 saturation 97% on room air.

Lab results:

Hemoglobin: 154 g/L (140-180)

Leukocytes: 17 x 10^9/L (3.5-12)

Platelets: 550 x 10^9/L (150-400)

Sodium: 126 mmol/L (135-145)

Potassium: 5.3 mmol/L (3.5-5.1)

Chloride: 88 mmol/L (96-106)

Bicarbonate: 8 mmol/L (3.9-6.1)

Serum glucose: 32 mmol/L (3.9-6.1)

Serum ketones: Positive (Negative)

Urine ketones positive

He is admitted for diabetic ketoacidosis and intravenous insulin infusion is started.

How often would you check Mr.C's electrolytes while he remains on IV insulin infusion for his diabetic ketoacidosis?

Every 2-4 hours

Every 4-8 hours

Every 8-12 hours

Daily

Submit

UNIVERSITY OF CALGARY
CUMMING SCHOOL OF MEDICINE

All content © 2020 University of Calgary

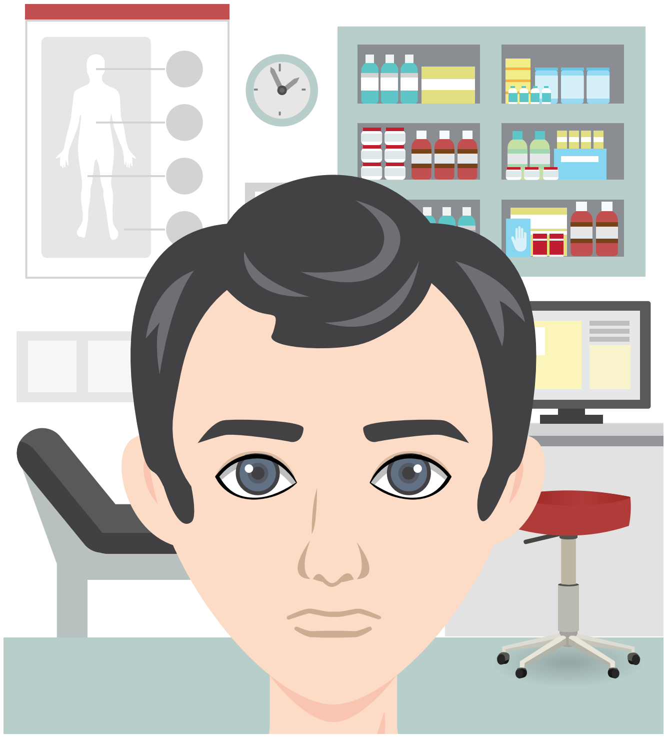

Mr. C is a 45 year old man with longstanding diabetes mellitus type 1 who presents to hospital with abdominal pain, vomiting, and elevated blood glucose readings at home.

Past medical history:

End-stage kidney disease secondary to diabetes, on 3X/week hemodialysis, no residual renal function

Peripheral Neuropathy

Hypertension

Dyslipidemia

Current medications: gabapentin 100mg tid, amlodipine 10mg daily, hydralazine 25 mg tid, rosuvastatin 5mg daily, polysaccharide iron complex 150 mg daily, darbepoetin alfa 30mcg/week

Examination: T36.5ºC, HR 130, RR 32, BP 100/64, O2 saturation 97% on room air.

Lab results:

Hemoglobin: 154 g/L (140-180)

Leukocytes: 17 x 10^9/L (3.5-12)

Platelets: 550 x 10^9/L (150-400)

Sodium: 126 mmol/L (135-145)

Potassium: 5.3 mmol/L (3.5-5.1)

Chloride: 88 mmol/L (96-106)

Bicarbonate: 8 mmol/L (3.9-6.1)

Serum glucose: 32 mmol/L (3.9-6.1)

Serum ketones: Positive (Negative)

Urine ketones positive

He is admitted for diabetic ketoacidosis and intravenous insulin infusion is started.

Update:

Mr C's ketoacidosis has resolved by the next day and he has successfully transitioned back to his home basal bolus insulin regimen.

You determine that his diabetic ketoacidosis was precipitated by a preceding viral gastroenteritis, which has resolved. Nephrology will arrange for dialysis for the patient while in hospital.

You anticipate being able to discharge the patient home within the next 24-48 hours.

How often does Mr. C need to have his creatinine checked in hospital?

Prior to the dialysis run only

Prior to the dialysis run and post-dialysis

Daily creatinine levels

No creatinine monitoring required

Submit

UNIVERSITY OF CALGARY
CUMMING SCHOOL OF MEDICINE

All content © 2020 University of Calgary

Cards (/)

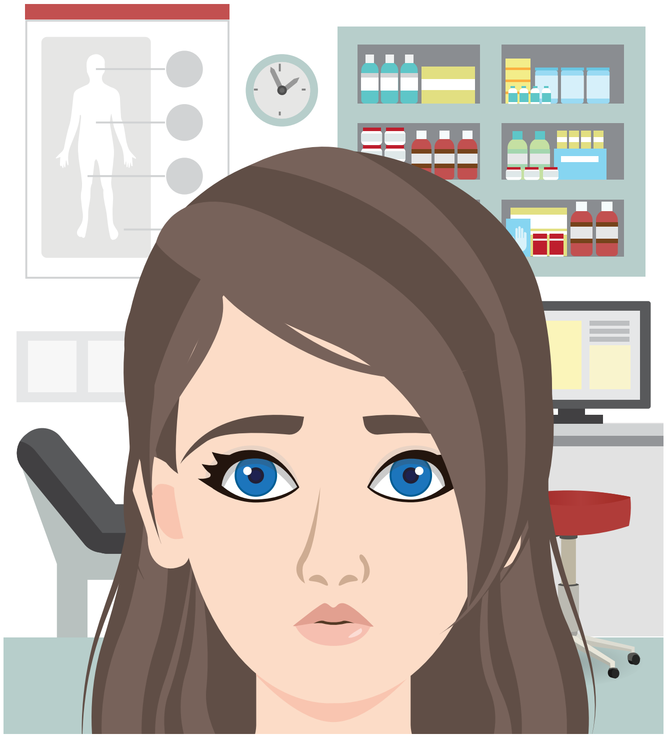

Ms D is a 24 year old woman who presents with a two day history of fevers, rigors and left flank pain.

Past medical history:

Prior chlamydia- fully treated.

Depression

Current medications: oral contraceptive pill, venlafaxine 75 mg daily, recent ibuprofen use for the fevers and flank pain

Examination: T40ºC, HR 110, RR 20, BP 80/40, O2 saturation 95% on room air. Left costovertebral angle tenderness is noted.

She is admitted for pyelonephritis and initiated on intravenous fluids and ceftriaxone.

Lab results:

Hemoglobin: 156 g/L (120-160)

Leukocytes: 20 x 10^9/L (3.5-12)

Platelets: 510 x 10^9/L (150-400)

Sodium: 147 mmol/L (135-145)

Potassium: 3.8 mmol/L (3.5-5.1)

Chloride: 97 mmol/L (96-106)

Bicarbonate: 24 mmol/L (3.9-6.1)

Creatinine: 54 mmol/L (3.9-6.1) GFR 128 Ml/min/$1.73m^2$

Urinalysis: positive for leukocyte esterase, nitrites, many white blood cells.

How often should Ms. D’s creatinine be checked in hospital?

No monitoring required since baseline is normal

Daily for the next few days

Twice a day for the next few days

Daily for the remainder of hospitalization

Submit

UNIVERSITY OF CALGARY
CUMMING SCHOOL OF MEDICINE

All content © 2020 University of Calgary

Cards (/)

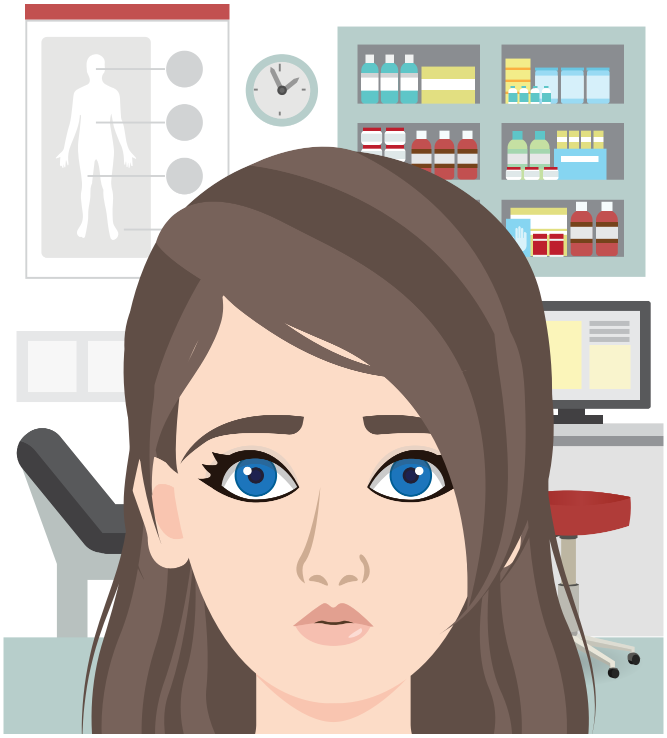

Ms D is a 24 year old woman who presents with a two day history of fevers, rigors and left flank pain.

Past medical history:

Prior chlamydia- fully treated.

Depression

Current medications: oral contraceptive pill, venlafaxine 75 mg daily, recent ibuprofen use for the fevers and flank pain

Examination: T40ºC, HR 110, RR 20, BP 80/40, O2 saturation 95% on room air. Left costovertebral angle tenderness is noted.

She is admitted for pyelonephritis and initiated on intravenous fluids and ceftriaxone.

Lab results:

Hemoglobin: 156 g/L (120-160)

Leukocytes: 20 x 10^9/L (3.5-12)

Platelets: 510 x 10^9/L (150-400)

Sodium: 147 mmol/L (135-145)

Potassium: 3.8 mmol/L (3.5-5.1)

Chloride: 97 mmol/L (96-106)

Bicarbonate: 24 mmol/L (3.9-6.1)

Creatinine: 54 mmol/L (3.9-6.1) GFR 128 Ml/min/$1.73m^2$

Urinalysis: positive for leukocyte esterase, nitrites, many white blood cells.

Update:

With treatment, Ms. D's flank pain, hypotension, and fevers resolve over the next two days. Her creatinine however rises to 80 µmol/L (from 54 µmol/L) on day 3 and to 100 µmol/L on day 4.

How often should we now be checking Ms. D's creatinine in hospital?

No more checks since this was an expected evolution

Daily for the next few days

Twice a day for the next three days

Daily for the remainder of the hospitalization

Submit

UNIVERSITY OF CALGARY
CUMMING SCHOOL OF MEDICINE

All content © 2020 University of Calgary

Cards (/)

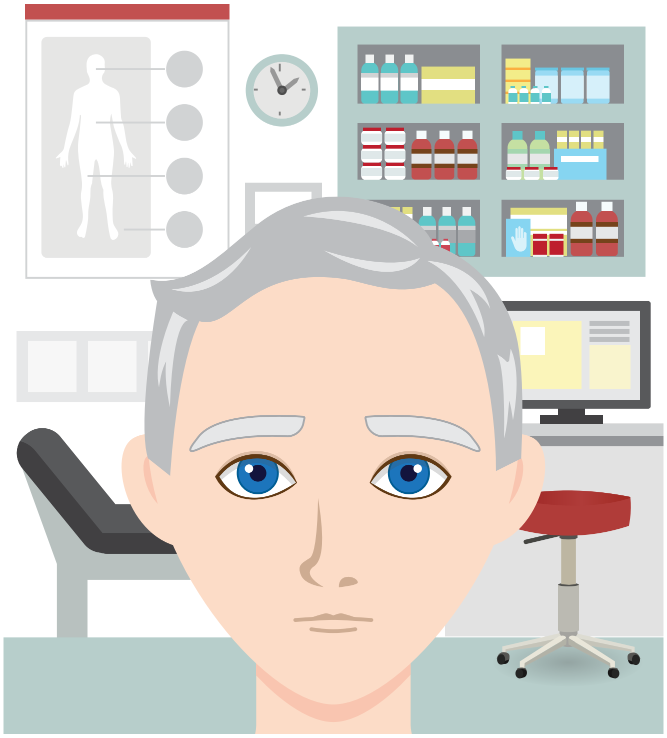

Mr E is a 77 year old gentleman who presents with 3 days of progressive dyspnea on exertion and pleuritic chest pain.

Past medical history:

Chronic kidney disease: baseline GFR 8 mL/min/1.73m2
Hypertension
Remote history of gastrointestinal bleeding due to multiple arteriovenous malformations

Current medications: amlodipine 10 mg daily, hydralazine 25mg qid, darbopoetin alfa 30cmg weekly, sodium bicarbonate 650mg tid

Examination: T38ºC, HR 110, RR 28, BP 120/70, O2 saturation 88% on room air. His physical exam is consistent with signs of right heart strain.

Lab results:

Hemoglobin: 145 g/L (140-180)

Leukocytes: 13 x 10^9/L (3.5-12)

Platelets: 380x 109/L (150-400)

Sodium: 137 mmol/L (135-145)

Potassium: 5.6 mmol/L (3.5-5.1)

Creatinine: 550 mmol/L (3.9-6.1) GFR 9 Ml/min/1.73m^2

Other tests:

Chest X-ray: Normal

D-dimer: High

EKG: Signs of right heart strain

VQ scan: High probability with segmental mismatched perfusion defects in the left lung

The patient is started on IV unfractionated heparin and is admitted to hospital for further management of his pulmonary embolism

Which of the following laboratory testing can be used to monitor his coagulation status while on intravenous unfractionated heparin? (Choose all that apply)

PTT based nomogram

INR based nomogram

Heparin anti-Xa level based nomogram

No monitoring required

Submit

UNIVERSITY OF CALGARY
CUMMING SCHOOL OF MEDICINE

All content © 2020 University of Calgary

Cards (/)

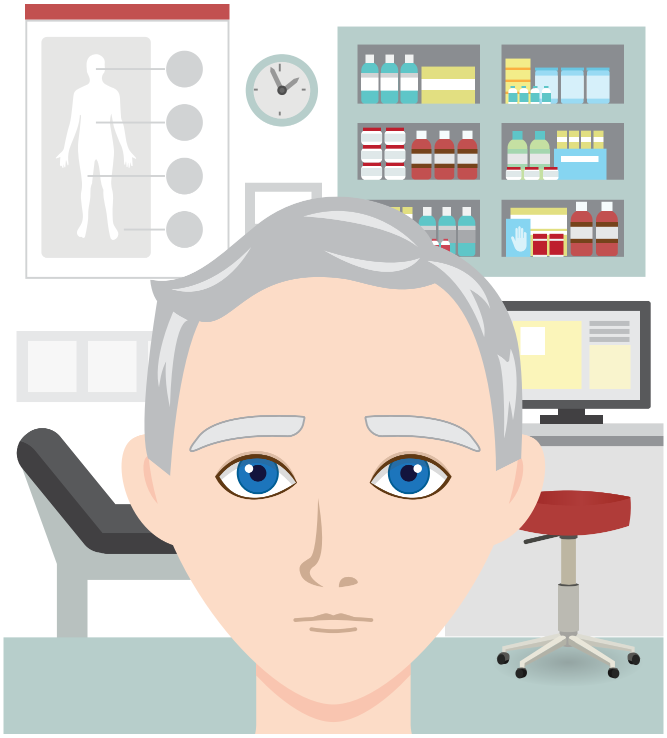

Mr E is a 77 year old gentleman who presents with 3 days of progressive dyspnea on exertion and pleuritic chest pain.

Past medical history:

Chronic kidney disease: baseline GFR 8 mL/min/1.73m2
Hypertension
Remote history of gastrointestinal bleeding due to multiple arteriovenous malformations

Current medications: amlodipine 10 mg daily, hydralazine 25mg qid, darbopoetin alfa 30cmg weekly, sodium bicarbonate 650mg tid

Examination: T38ºC, HR 110, RR 28, BP 120/70, O2 saturation 88% on room air. His physical exam is consistent with signs of right heart strain.

Lab results:

Hemoglobin: 145 g/L (140-180)

Leukocytes: 13 x 10^9/L (3.5-12)

Platelets: 380x 109/L (150-400)

Sodium: 137 mmol/L (135-145)

Potassium: 5.6 mmol/L (3.5-5.1)

Creatinine: 550 mmol/L (3.9-6.1) GFR 9 Ml/min/1.73m^2

Other tests:

Chest X-ray: Normal

D-dimer: High

EKG: Signs of right heart strain

VQ scan: High probability with segmental mismatched perfusion defects in the left lung

The patient is started on IV unfractionated heparin and is admitted to hospital for further management of his pulmonary embolism

Update:

Mr. E is transitioned from heparin to warfarin and he is no longer requiring oxygen. He is working with physiotherapy to improve his strength and mobility.

He has developed no new issues in hospital and has resumed his home medications with the only new medication being warfarin. His INRs are now in the therapeutic range on a stable dose.

You are planning for discharge in the next two days. His renal function and hyperkalemia so far in hospital have remained stable.

What bloodwork would you order on Mr. E during the remainder of his hospital stay?

Daily CBC, electrolytes and creatinine

Daily electrolytes

Daily creatinine

CBC, electrolytes and creatinine on the day of discharge only

Submit

UNIVERSITY OF CALGARY
CUMMING SCHOOL OF MEDICINE

All content © 2020 University of Calgary

Cards (/)

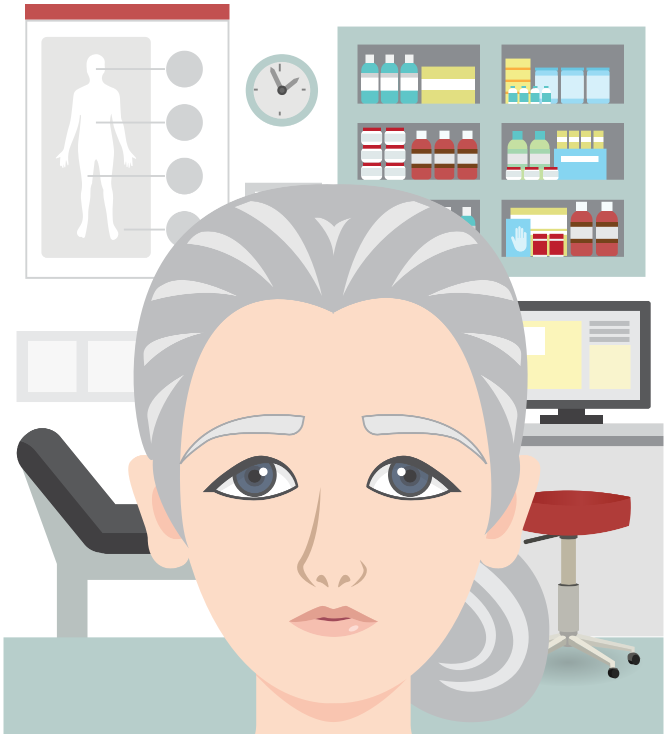

Mrs. F is a 90 year old woman who presents to the hospital with fevers, fatigue, new left leg pain and unable to ambulate independently at home.

Past medical history:

Prior known colonization with community-acquired MRSA

Moderate aortic stenosis

Osteoporosis

Mild cognitive impairment

Current medications: alendronate 70mg weekly, calcium and vitamin D.

Examination: T38.5ºC, HR 110, RR 18, BP 120/70, O2 saturation 93% on room air. Her physical exam is consistent with signs of left leg cellulitis.

Lab results:

Hemoglobin: 120 g/L (120-160)

Leukocytes: 18 x 10^9/L (3.5-12)

Platelets: 425 x 109/L (150-400)

Sodium: 137 mmol/L (135-145)

Potassium: 4.0 mmol/L (3.5-5.1)

Creatinine: 150 µmol/L (3.9-6.1) Prior results 60 µmol/L

She is admitted for cellulitis and started on treatment with cefazolin.

What daily bloodwork for the next 3 days will you order for Ms. F at admission?

CBC

CBC and electrolytes

CBC, electrolytes, and creatinine

CBC, electrolytes, creatinine and urea

Submit

UNIVERSITY OF CALGARY
CUMMING SCHOOL OF MEDICINE

All content © 2020 University of Calgary

Cards (/)

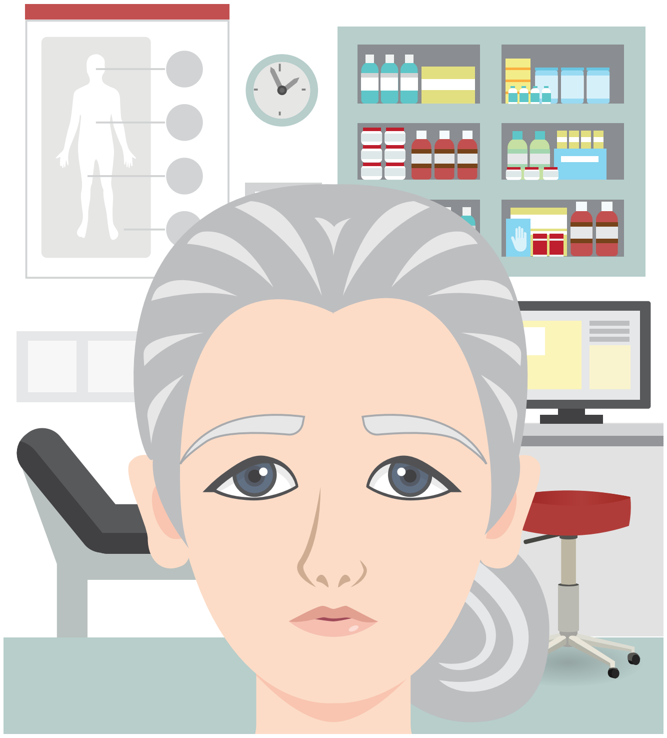

Mrs. F is a 90 year old woman who presents to the hospital with fevers, fatigue, new left leg pain and unable to ambulate independently at home.

Past medical history:

Prior known colonization with community-acquired MRSA

Moderate aortic stenosis

Osteoporosis

Mild cognitive impairment

Current medications: alendronate 70mg weekly, calcium and vitamin D.

Examination: T38.5ºC, HR 110, RR 18, BP 120/70, O2 saturation 93% on room air. Her physical exam is consistent with signs of left leg cellulitis.

Lab results:

Hemoglobin: 120 g/L (120-160)

Leukocytes: 18 x 10^9/L (3.5-12)

Platelets: 425 x 109/L (150-400)

Sodium: 137 mmol/L (135-145)

Potassium: 4.0 mmol/L (3.5-5.1)

Creatinine: 150 µmol/L (3.9-6.1) Prior results 60 µmol/L

She is admitted for cellulitis and started on treatment with cefazolin.

Update:

Mr. F’s cellulitis and acute kidney injury resolve over the next few days.

Upon further assessment from physiotherapy and occupational therapy, and in discussion with patient and family, the decision is made that Ms. F should be discharged to a long-term care center.

She will likely remain in hospital for the next few weeks until a long-term care bed is available

How often should Mrs. F’s CBC, electrolytes, creatinine be monitored in hospital?

Every 2 days

Every 3 days

Weekly

Decide based on daily history, physical exam, and clinical assessment

Submit

UNIVERSITY OF CALGARY
CUMMING SCHOOL OF MEDICINE

All content © 2020 University of Calgary

Cards (/)

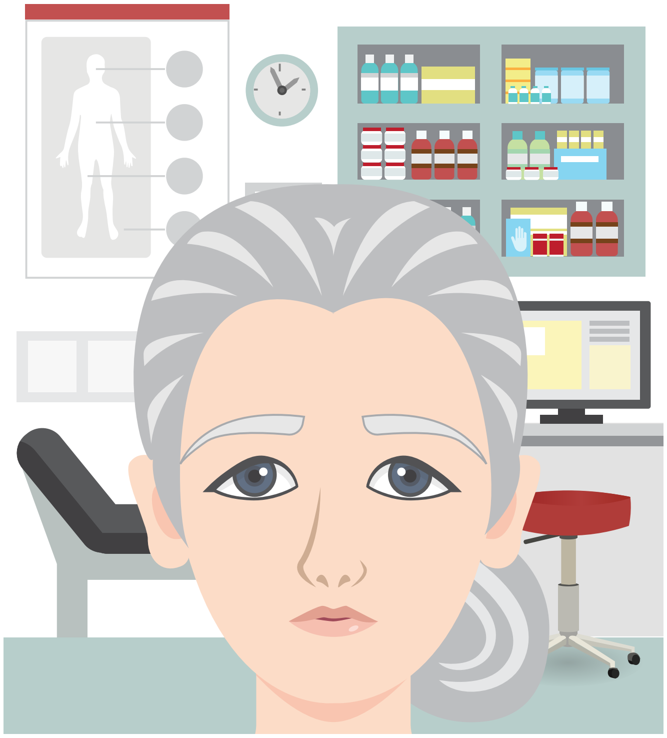

HR	70	bpm
SpO2	98	%
RR	13	BrPM
Temp	36.6	°C
NBP	121/78	mmHg

Mrs. F is a 90 year old woman who presents to the hospital with fevers, fatigue, new left leg pain and unable to ambulate independently at home.

Past medical history:

Prior known colonization with community-acquired MRSA

Moderate aortic stenosis

Osteoporosis

Mild cognitive impairment

Current medications: alendronate 70mg weekly, calcium and vitamin D.

Examination: T38.5ºC, HR 110, RR 18, BP 120/70, O2 saturation 93% on room air. Her physical exam is consistent with signs of left leg cellulitis.

Lab results:

Hemoglobin: 120 g/L (120-160)

Leukocytes: 18 x 10^9/L (3.5-12)

Platelets: 425 x 109/L (150-400)

Sodium: 137 mmol/L (135-145)

Potassium: 4.0 mmol/L (3.5-5.1)

Creatinine: 150 µmol/L (3.9-6.1) Prior results 60 µmol/L

She is admitted for cellulitis and started on treatment with cefazolin.

Update:

Mr. F's cellulitis and acute kidney injury resolve over the next few days.

Upon further assessment from physiotherapy and occupational therapy, and in discussion with patient and family, the decision is made that Ms. F should be discharged to a long-term care center.

She will likely remain in hospital for the next few weeks until a long-term care bed is available

Update:

Ms. F remains stable for the next week under your care

On day 20 of her admission she reports new diarrhea that is corroborated by the nursing staff. On physical examination, she appears more lethargic and hypovolemic. You are concerned about possible C.difficile diarrhea. Stool sample for C.difficile toxin is pending. Your initial bloodwork now comes back with an elevated white blood cell count of 30 X 10^9/L.

How often should we monitor the CBC in this patient?

No more testing required

Weekly

Daily for the next few days then reassess

Twice daily until trending downwards

Submit

UNIVERSITY OF CALGARY
CUMMING SCHOOL OF MEDICINE

All content © 2020 University of Calgary

Cards (/)

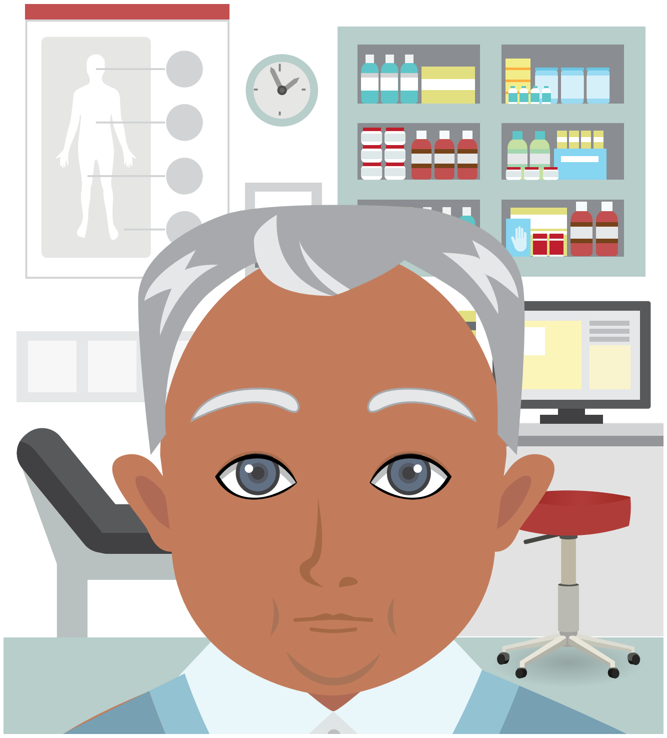

Mr G is an 80 year old man coming in with severe fatigue, and new complaints of easy bruising and bleeding.

Past medical history:

Diabetes Mellitus II with neuropathy
Coronary artery disease with prior percutaneous coronary intervention 5 years ago
Hypertension
Moderate chronic obstructive pulmonary disease

Current medications: metformin 500mg tid , pregabalin 150mg per day, ASA 81g daily, rosuvastatin 20mg daily, perindopril 4mg daily , tiotropium 18 mcg inhaled daily, Fluticasone/salmeterol 250/50 1 puff bid

Examination: T38ºC, HR 110, RR 22, BP 113/78, O2 saturation 91% on room air. You note pallor and petechiae and ecchymoses. You also palpate marked lymphadenopathy in his cervical and bilateral supraclavicular chains.

In addition to complete blood count, what additional investigations would you initially order to work up Mr. G's pallor, petechiae and ecchymoses?

No additional testing needed

Peripheral smear

Peripheral smear and INR

Peripheral smear, INR and PTT

Submit

All content © 2020 University of Calgary

Cards (/)

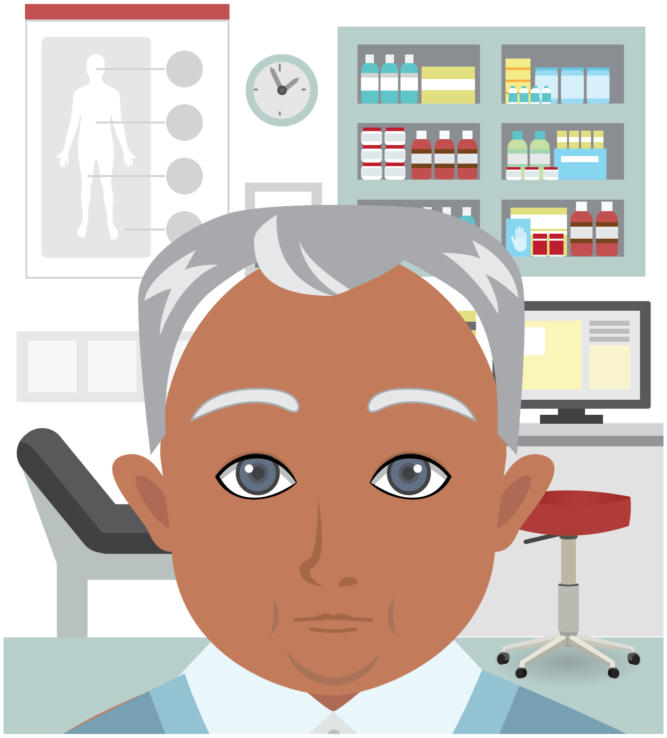

Mr G is an 80 year old man coming in with severe fatigue, and new complaints of easy bruising and bleeding.

Past medical history:

Diabetes Mellitus II with neuropathy
Coronary artery disease with prior percutaneous coronary intervention 5 years ago
Hypertension
Moderate chronic obstructive pulmonary disease

Current medications: metformin 500mg tid , pregabalin 150mg per day, ASA 81g daily, rosuvastatin 20mg daily, perindopril 4mg daily , tiotropium 18 mcg inhaled daily, Fluticasone/salmeterol 250/50 1 puff bid

Examination: T38ºC, HR 110, RR 22, BP 113/78, O2 saturation 91% on room air. You note pallor and petechiae and ecchymoses. You also palpate marked lymphadenopathy in his cervical and bilateral supraclavicular chains.

Update:

Mr. G's initial laboratory investigations show a hemoglobin 65g/L, WBC 10 X 10^9/L, platelets 49 X 10^9/L, normal PTT and INR, and a smear showing polychromasia, reticulocytosis, and decreased platelets. With the assistance of other laboratory testing and hematology advice, you establish a diagnosis of autoimmune hemolytic anemia (AIHA) and immune thrombocytopenic purpura (ITP).

How often does Mr. G need his CBC monitored in hospital

Every 2-4 hours

Every 6-12 hours

Daily x 3 days then reassess

Only if he develops overt clinical bleeding

Submit

UNIVERSITY OF CALGARY
CUMMING SCHOOL OF MEDICINE

All content © 2020 University of Calgary

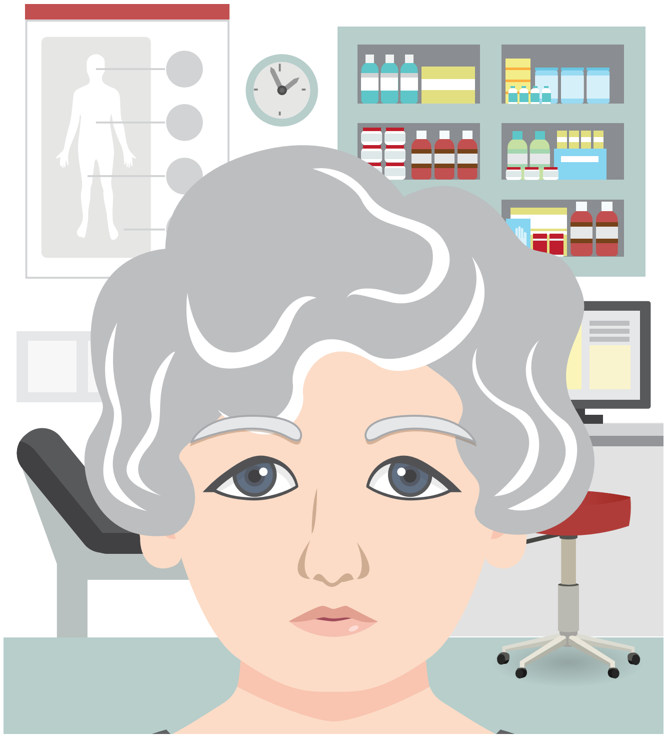

Mrs. H is a 69 year old woman who presents with fever, headache and confusion.

Past medical history:

Hypertension

Obstructive sleep apnea

Prior right total knee replacement

Current medications: perindopril 8mg daily.

Examination: T39.5ºC, HR 110, RR 18, BP 120/70, O2 saturation 93% on room air. Her physical exam shows nuchal rigidity

Lab results:

Hemoglobin: 120 g/L (120-160)

Leukocytes: 23 x 10^9/L (3.5-12)

Platelets: 370 x 109/L (150-400)

Sodium: 137 mmol/L (135-145)

Potassium: 4.5 mmol/L (3.5-5.1)

Creatinine: 79 µmol/L (50-110) Prior results 60 µmol/L

CT head: no acute findings, no mass lesions identified

Due to concern regarding meningitis, a lumbar puncture is arranged.

What additional laboratory testing should you perform prior to the lumbar puncture?

No additional testing required

INR

PTT

INR and PTT

Submit

UNIVERSITY OF CALGARY
CUMMING SCHOOL OF MEDICINE

All content © 2020 University of Calgary

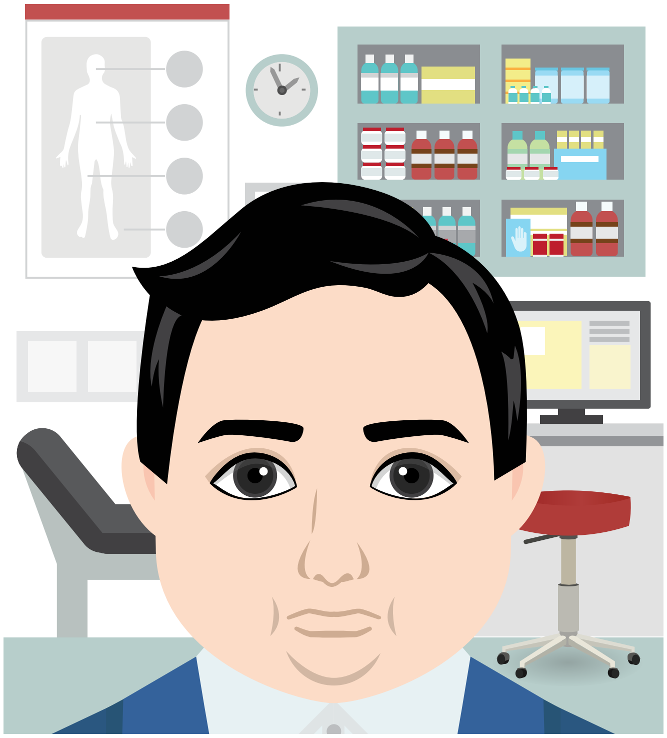

The following questions are an opportunity to test your knowledge

In which of the following cases might it be useful to order a urea level?

To calculate osmolar gap in a patient with suspected toxic alcohol ingestion

To monitor renal status in patients with acute renal failure

To decide when to initiate renal replacement therapy in patients with renal failure

All of the above

Submit

All content © 2020 University of Calgary

Cards (/)

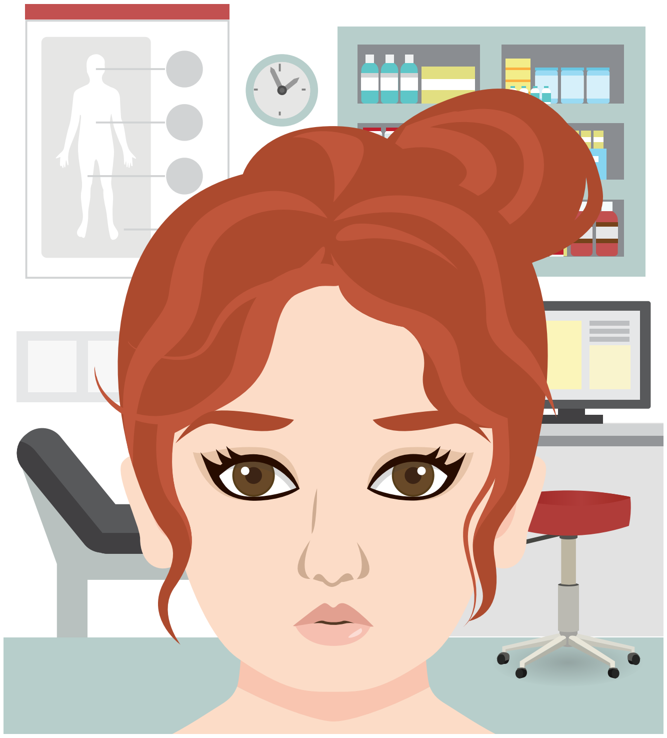

The following questions are an opportunity to test your knowledge

What is the optimal frequency of monitoring electrolytes and acid-base status in patient just admitted with diabetic ketoacidosis on intravenous insulin infusion?

Every hour until the anion gap has resolved

Every 2-4 hours until anion gap has resolved

Every 2-4 hours X 48 hours regardless of anion gap

Every 6-8 hours until the patient is off the IV insulin infusion

Submit

UNIVERSITY OF CALGARY
CUMMING SCHOOL OF MEDICINE

All content © 2020 University of Calgary

Cards (/)

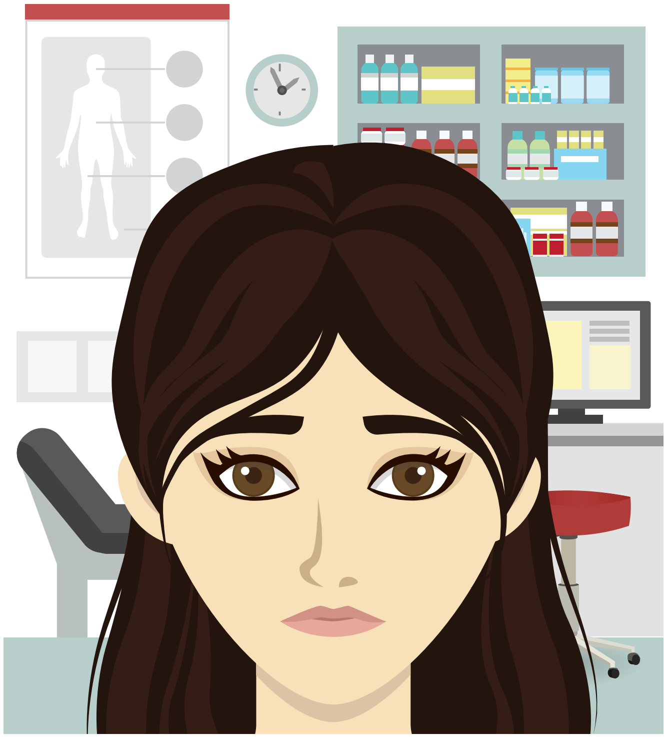

The following questions are an opportunity to test your knowledge

What panel of routine laboratory tests should be used to monitor stable inpatients awaiting rehabilitation, transition and/or placement ?

Complete blood count

Complete blood count, creatinine and electrolytes

Complete blood count, creatinine, urea, and electrolytes

No specific panel of routine laboratory tests

Submit

UNIVERSITY OF CALGARY
CUMMING SCHOOL OF MEDICINE

All content © 2020 University of Calgary

Cards (/)

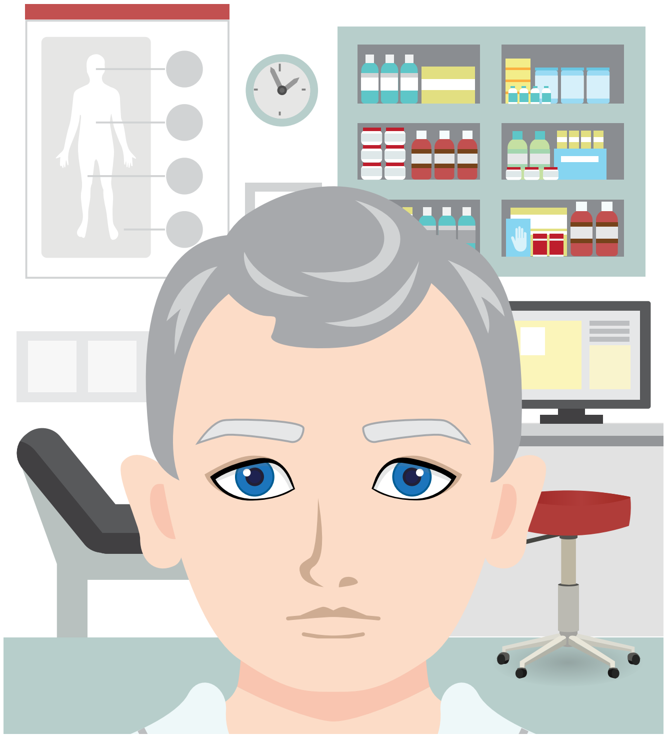

An 78 year old man with anuria on intermittent hemodialysis was admitted to your team with fevers, rigors and new leukocytosis. His blood cultures return back with gram negative bacilli and based on prior susceptibility data on this patient you start him on appropriately dosed gentamicin. He remains hemodynamically stable and is on appropriate renally-dosed antimicrobial coverage. You have consulted nephrology to manage his hemodialysis.

You decide to closely monitor this patient for the next 2-3 days by ordering few laboratory tests. Which tests would you order?

Daily CBC only

Daily CBC and electrolytes

Daily CBC, electrolytes and creatinine

Daily CBC, electrolytes, creatinine and urea

Submit

All content © 2020 University of Calgary

Cards (/)

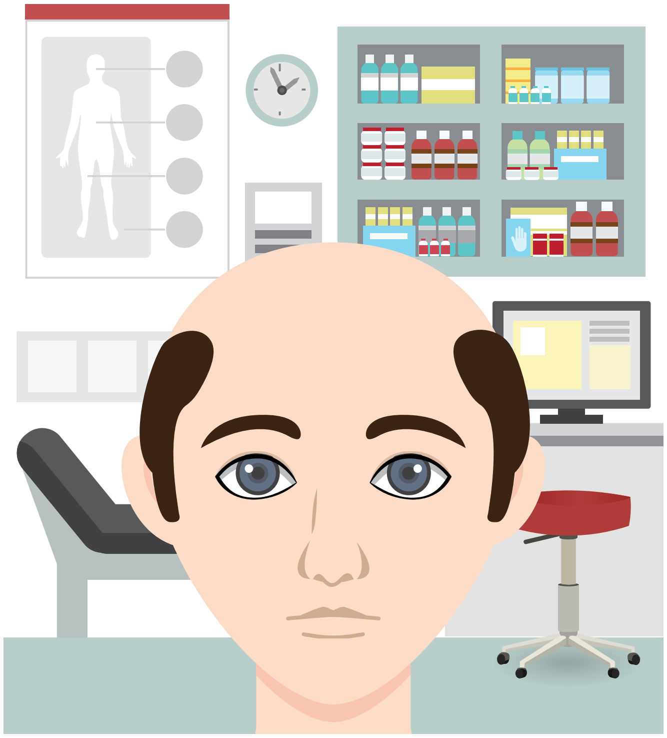

The following questions are an opportunity to test your knowledge

In which of the following scenarios would it be reasonable to order a PTT?

In a patient presenting with new complaints of easy bruising and bleeding

In a patient on an infusion of unfractionated heparin for an acute coronary syndrome

In a patient suspected to have DIC

All of the above

None of the above

Submit

UNIVERSITY OF CALGARY
CUMMING SCHOOL OF MEDICINE

All content © 2020 University of Calgary

Cards (/)

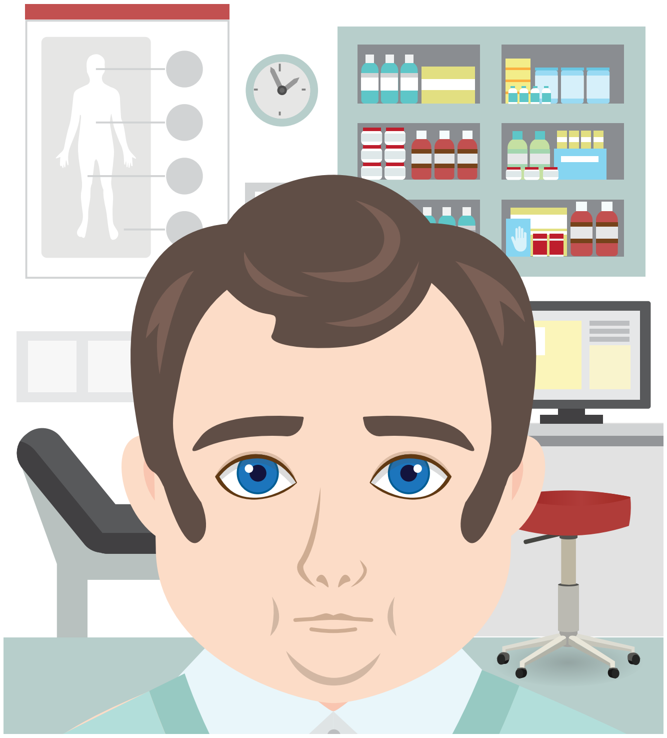

You are taking care of patient. Patient was admitted seven days ago with pneumonia. He has improved clinically with antibiotics. You notice today that he has developed new severe thrombocytopenia in hospital with platelets of 49 today. You review his chart and note that in addition to the antibiotics he was also on unfractionated heparin subcutaneously for DVT prophylaxis.

In addition to discontinuing potential offending drugs and initiating the appropriate workup, how often should you monitor his platelets?

No more CBC testing required

Every other day

Check once in a few days

Daily for the next 3 days

Every 12 hours for the next 3 days

Submit

UNIVERSITY OF CALGARY
CUMMING SCHOOL OF MEDICINE

All content © 2020 University of Calgary

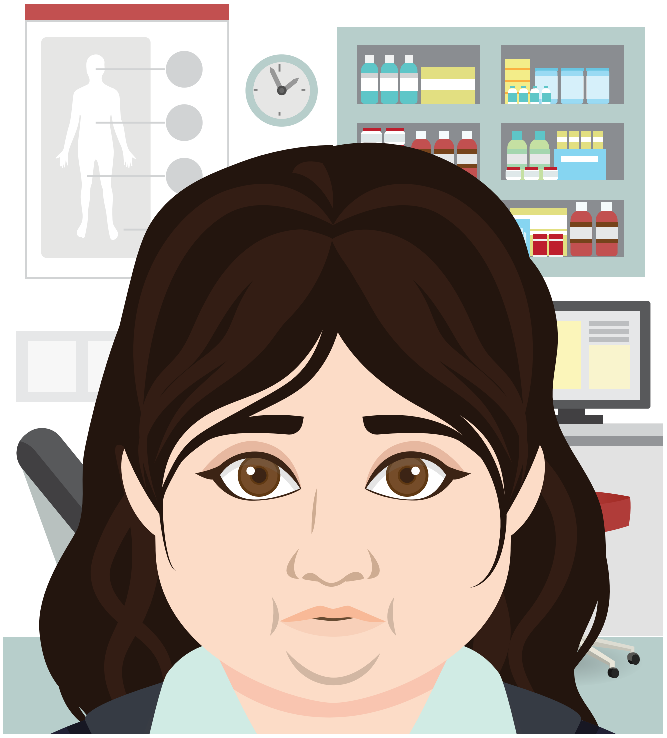

You admitted a 45 year old woman to your team last night with an acute kidney injury and creatinine of 190 μmol/L (baseline of 65 μmol/L). Today her creatinine has increased to 240 μmol/L.

As you investigate this patient for different causes of acute kidney injury, how often should you be checking her creatinine in hospital?

Twice a week

Daily for the next 3 days

Every 12 hours for the next 3 days

Every 6 hours until creatinine starts to improve

Submit

UNIVERSITY OF CALGARY
CUMMING SCHOOL OF MEDICINE

All content © 2020 University of Calgary

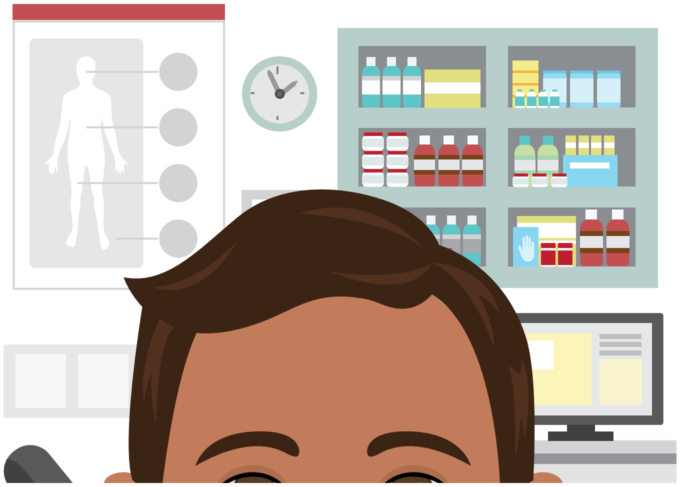

Thanks so much for completing this deck.

See below for the summary of content and a link to provide feedback on this module.

Summary of key messages:

- Stable inpatients awaiting rehabilitation/transition and/or placement generally do not require routine testing of complete blood count, electrolytes and creatinine unless driven by specific diagnostic suspicion
- Urea testing is not indicated for most stable inpatients without specific diagnostic suspicion
- In a newly admitted patient with a new diagnosis of severe anemia (hemoglobin < 70g/L) without overt bleeding, complete blood count should be monitored daily initially
- In a patient being worked up for new leukocytosis, daily testing of complete blood count is generally sufficient and should be re-evaluated within 3 days
- In a patient with new severe thrombocytopenia (platelets <50 X 109/L), complete blood count should be monitored daily initially
- In a patient with diabetic ketoacidosis requiring intravenous insulin infusion for treatment, serum electrolytes should be monitored every 2-4 hours
- In a patient on new medications/treatments associated with electrolyte abnormalities, initially electrolytes should be monitored daily
- In a patient with stable hyperkalemia (5.5-6.0 mmol/L) no more than daily monitoring of potassium levels in the inpatient setting is sufficient
- In a patient admitted with sepsis, creatinine should be monitored daily initially
- In a patient on new nephrotoxic agents/therapies (e.g. contrast dye, diuretics, antibiotics, etc.) initially creatinine should be monitored daily
- In a patient with acute worsening renal function, creatinine should be monitored on a daily basis initially
- In a patient on chronic dialysis with no residual renal function, generally creatinine checks are not recommended unless there is a specific diagnostic question
- In a patient on intravenous heparin infusion, PTT or heparin Anti-Xa levels should be monitored per nomogram to ensure appropriate anticoagulation
- Partial thromboplastin time may be checked once to help diagnose a suspected bleeding diathesis
- INR should be tested prior to invasive procedures that recommend INR check based on patient risk factors and bleeding risk associated with the procedure

You've now completed this module.

You can fill out the following survey to provide feedback: link (https://survey.ucalgary.ca/jfe/form/SV_56ihu0iBr3nzxNX)

Here are some instructions on how to file for credits: Visit Mainport https://mainport.royalcollege.ca (https://mainport.royalcollege.ca) to record your learning and outcomes. If you are a CCFP member, you can claim 6 credits for Linking Learning to Assessment or 5 credits for Linking Learning to Practice or Administration. Here are instructions on how you can complete a Linking learning to

assessment exercise: Click Here To View
(https://cumming.ucalgary.ca/sites/default/files/teams/4/Preceptor%20Resources/Linking%20Learning%20to%20Assessment_Practice%

Please enter your name, email and institution below for a CME certificate of attendance.

255

Submit

UNIVERSITY OF CALGARY
CUMMING SCHOOL OF MEDICINE

All content © 2020 University of Calgary

1b. Clinical Decision Support Tool

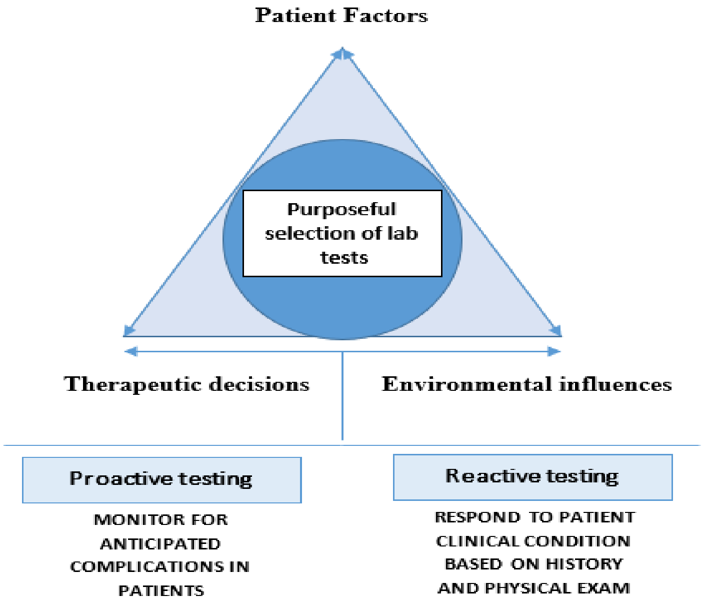

Laboratory Test	General Indications	Frequency Recommendations	Costs (in CAD)
Complete Blood Count	Active Bleeding<br>Acute infection/inflammation<br>Abnormal cell lines<br>Stable patient w/o indications	q2-8h<br>Daily<br>Daily or less<br>None	7.00
Electrolytes (Na, K, Cl, CO_2)	DKA with gap<br>Severe Na or K derangement<br>Chronic hyponatremia (<120)<br>Chronic hyponatremia (120-134)<br>Hypernatremia needing IVF<br>Stable hyperkalemia<br>New lowK requiring replacement<br>Stable patient w/o indications	q2-4h<br>q2-8h<br>Daily or more<br>Daily or less<br>Q6h-daily<br>Daily<br>BID-Daily<br>None	5.00 (Total or Each)
Creatinine	AKI<br>Nephrotoxic agents<br>Sepsis<br>Chronic Hemodialysis<br>Stable chronic kidney disease<br>Stable patient w/o indications	Daily or less<br>Daily<br>Daily<br>None<br>None<br>None	5.00

Laboratory Test	General Indications	Frequency Recommendations	Costs (in CAD)
Urea	GI Bleed, Osmolar Gap for Acute Intoxication or Metabolic Acidosis Syndrome<br>Renal Failure: Pre-Renal AKI vs. ATN, Adrenal Insufficiency<br>Pericarditis, Acute Pancreatitis, Hemolytic Ureic Syndrome<br>Community Acquired Pneumonia, Sickle Cell Anemia<br>Urea Clearance Testing for Dialysis, Severe Sepsis or Shock, Toxic Shock Syndrome<br>Uremic encephalopathy<br>Stable patient w/o indications	Once<br>Once<br>Once<br>Once<br>Once<br>None<br>None	7.50
Coagulation (PT/INR)	Bleeding diathesis<br>New start/dose warfarin<br>Stable dose warfarin<br>Prior to invasive procedures<br>Stable patient w/o indications	Depends on type of diathesis<br>Daily<br>q2-3days- weekly<br>Once if normal<br>No more than once<br>Note: NO daily INRs for stable INR on stable warfarin dose	7.50
Coagulation (PTT)	IV Heparin infusion<br>Diagnosing a Bleeding diathesis<br>Stable patient w/o indications	Per Protocol<br>Once<br>No more than once	7.50

2. Audit and Feedback

This is an example of the PowerPoint used to conduct the audit and feedback session over zoom.

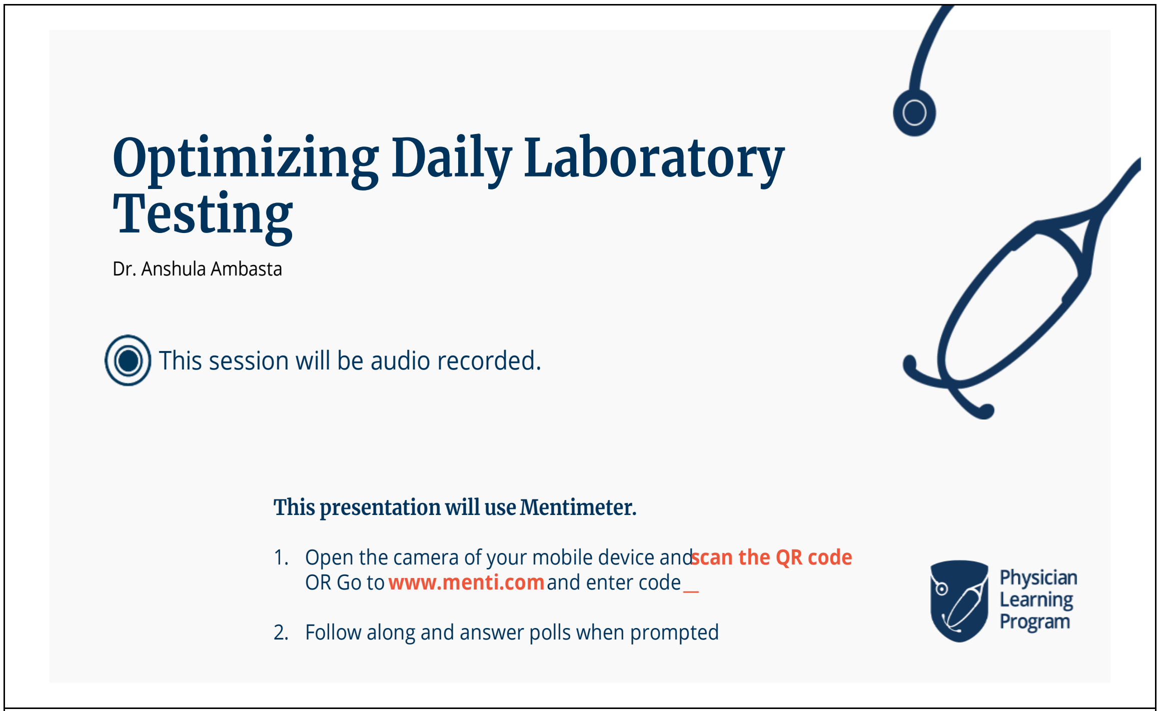

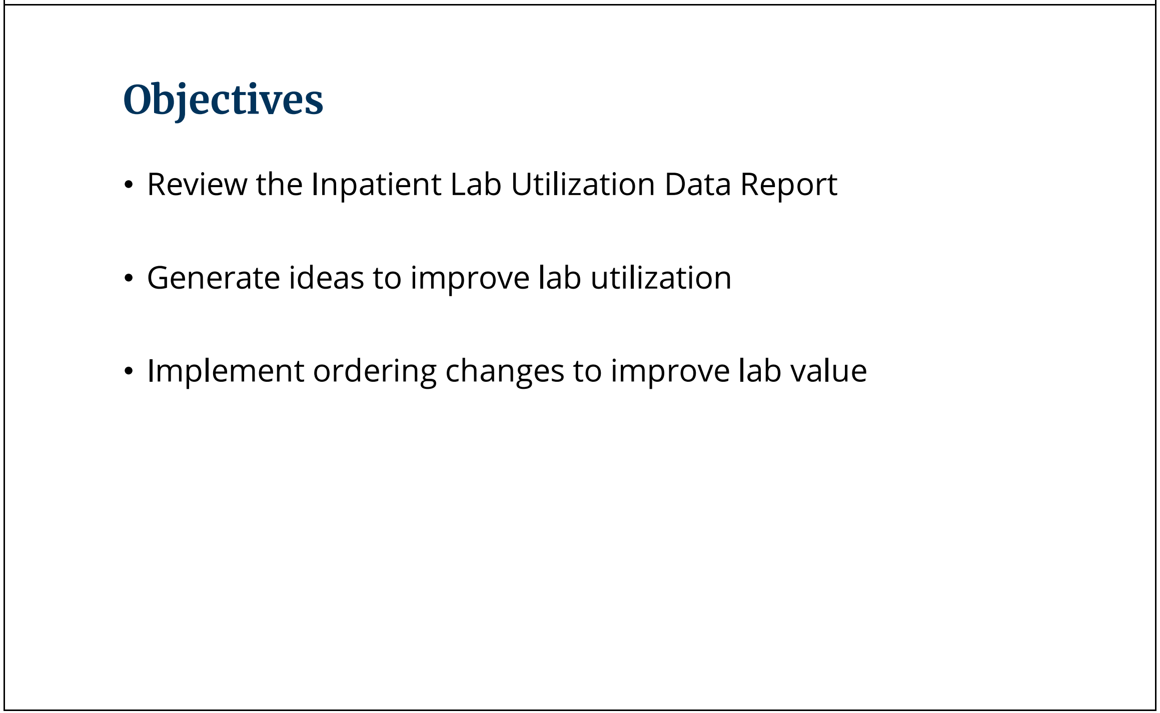

Why is it important to improve lab ordering?

Case Study

Patient:

Ms. B
Age: 90
Female

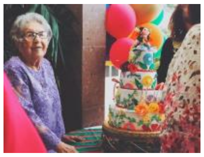

Ms. B is admitted for heart failure and treated with an initial intravenous bolus dose of furosemide, followed by daily diuretic therapy.

What laboratory tests would you order on Ms. B on Day 3 of her hospital admission?

What might patients want us to consider when we order laboratory tests?

- *"The entire process and timing is based on the routines in the hospital. It is not taking into consideration the needs and situation that particular patient is in. And it starts the process off on the wrong foot unfortunately…They flip on the lights particularly in the morning and wake him up when he had just fallen asleep…." Participant #1 (Family Member)*

- *"Please tell me what you suspect. If you suspect a urinary tract infection, tell me that. Tell me that's why you are taking the blood test…we're going to have to do it again because we're monitoring the changes... Now you know it's coming; you know what to anticipate." Participant #2 (Patient)*

Background

- Laboratory test ordering is the most common medical activity performed in the Canadian healthcare system [1]

- Over 20% of laboratory testing is considered wasteful[2]

- Canadians receive over 1 million unnecessary tests each year [3]

[1] Naugler C, Wyonch, R. What the Doctor Ordered: Improving the Use and Value of Laboratory Testing2019 October 4, 2019. Available from: https://www.cdhowe.org/public-policy-research/what-doctor-ordered-improving-use-and-value-laboratory-testing.
[2] Zhi M, Ding EL, Theisen-Toupal J, Whelan J, Arnaout R. The landscape of inappropriate laboratory testing: a 15-year meta-analysis. PloS one. 2013;8(11):e78962.
[3] Canadian Institute for Health Information Unnecessary Health Care in Canada. Unnecessary Health Care in Canada 2017 [cited 2018 April 17]. Available from:https://www.cihi.ca/en/unnecessary-care-in-canada-infographic.

Repetitive routine blood testing in hospitals is considered low value care

Internal Medicine

4. In the inpatient setting, don't order repeated CBC and chemistry testing in the face of clinical and lab stability.

Residents

1. Don't order investigations that will not change your patient's management plan.
2. Don't order repeat laboratory investigations on inpatients who are clinically stable.

Medical Students

4. Don't hesitate to ask for clarification on tests, treatments, or procedures that you believe are unnecessary.

Choosing Wisely Canada™

Significant Variation in MD Lab Ordering

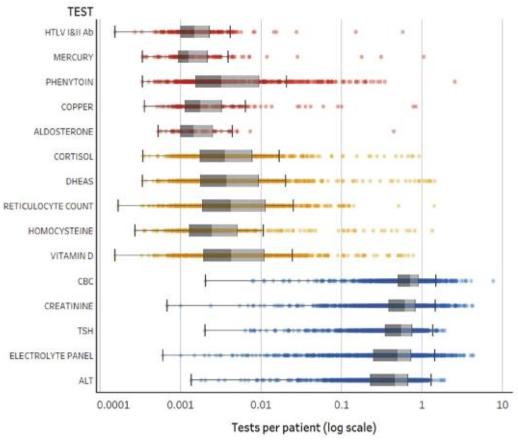

Nguyen LT, Guo M, Hemmelgarn B, Quan H, Clement F, Sajobi T, Thomas R, Turin TC, Naugler C. Evaluating practice variance among family physicians to identify targets for laboratory utilization management. Clinica Chimica Acta. 2019 Oct 1;497:1-5.

Does Audit and Feedback Work?

THE COCHRANE COLLABORATION®

SCAN ME

- **140 Clinical Trials**
- **A&F improves compliance with desired professional behavior by 4% (IQR 0.5-16%)**
- **A&F is more effective when . . .**
  - The source is a respected colleague
  - It is delivered both verbally and in written form
  - It is provided more than once
  - It includes explicit targets and an action plan

Source: Ivers N, et al. Cochrane Database of Systematic Reviews 2012; 6: CD000259.

Physician Learning Program

Evidence from Calgary Pilot

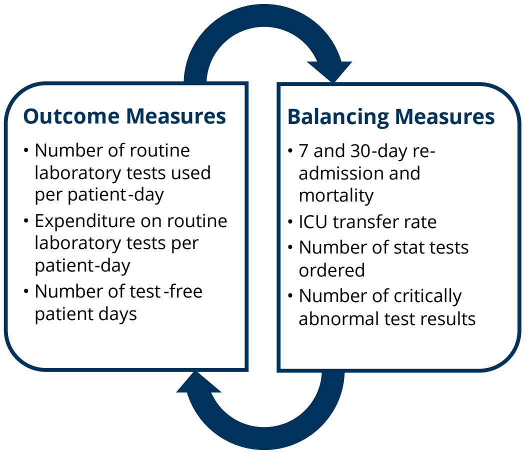

Evidence from our prior QI efforts

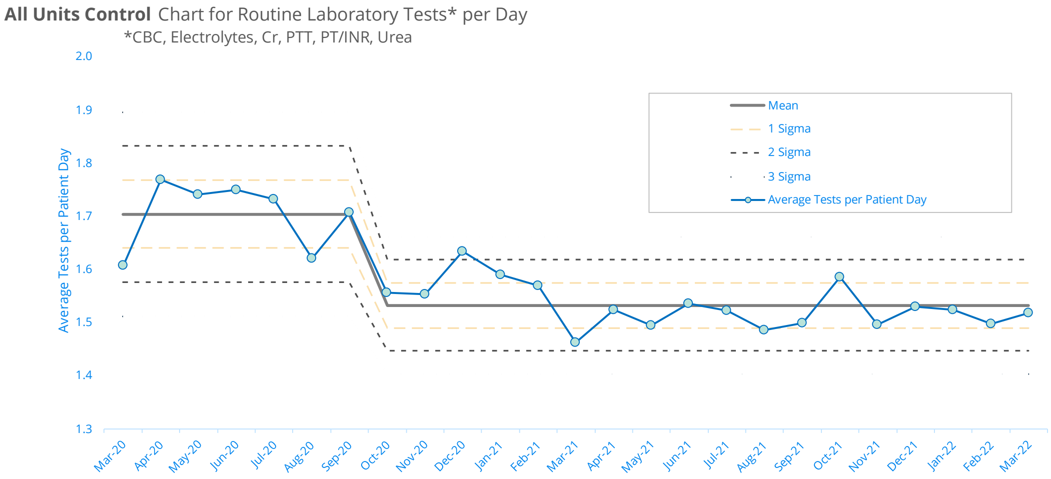

BMJ Qual Saf. 2023 May 10:bmjqs -2022-015611. doi: 10.1136/bmjqs -2022-015611

- The graph shows the monthly average routine test counts for .... Y-axis values are the resulting routine test per patient day for each unit. The mean value indicates the average of the monthly averages of all routine test count per patient day across all units of the site.

- Routine tests per patient day=sum of all routine tests/(number of unit beds*number of days in a month).

Insert graph here showing ordering patterns of the site

Issues with the data . . .

Making changes might be dangerous for my patients . . .

Multiple providers.

But I once had a patient that really needed that test!

My patients are different.

Who has access? The college? My patients?

What if the data makes me look bad?

This data is wrong.

Why do I need this? I know my habits already.

Rules

- You must make a change!
- Data has limitations
- There are no right or wrong ideas
- Everyone must participate
- Focus on what is in your control

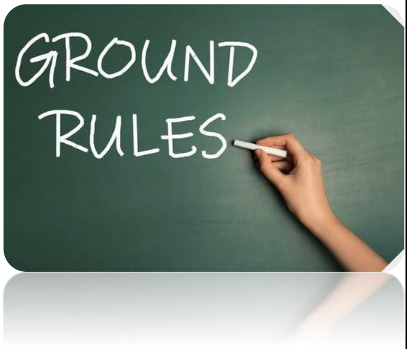

Brainstorming . . .

"No great mind has ever existed without a touch of madness"

Q: How could I increase the value of my lab ordering?
Write down as many ideas as you can in 3 minutes of silence.

Discussion

- What are your ideas? – Everyone shares 1 idea
- Try to not repeat something that was already mentioned
- Think out of the box
- No judgement, no ranking

How could you measure high value lab ordering?

- Menti word cloud

Prioritizing Ideas

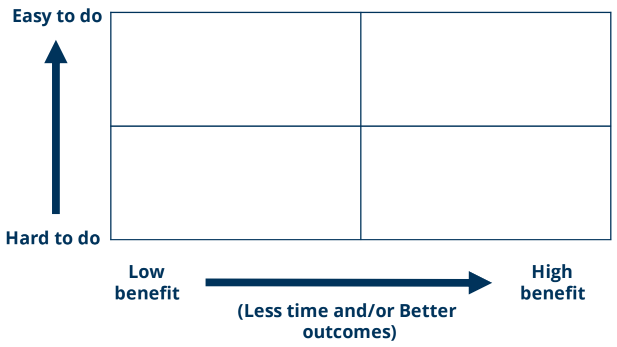

Commitment to Change

Take a few minutes to answer the questions on the form:

https://survey.ucalgary.ca/jfe/form/...

CME Credits: Claiming Royal College Section 3 Credits

Optimization of Laboratory Test Utilization Amongst Inpatients

Info regarding CME credits

Evaluation

Workshop Feedback Survey

Please take a few minutes and let us know how the session went today:

...

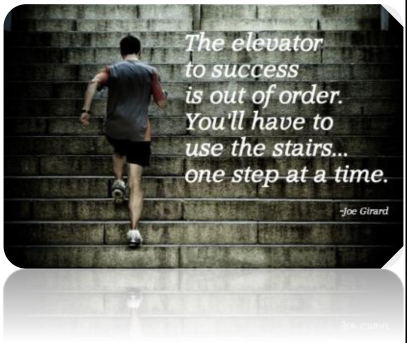

3. **EMR Ordering Process Changes**

Limit usage of daily repeating orders
Use of every 2-3 days or Monday, Wednesday, Friday blood work testing
Use of add-on options from previously drawn bloodwork
Reviewing orders at hand-offs

4a. Patient Infographic

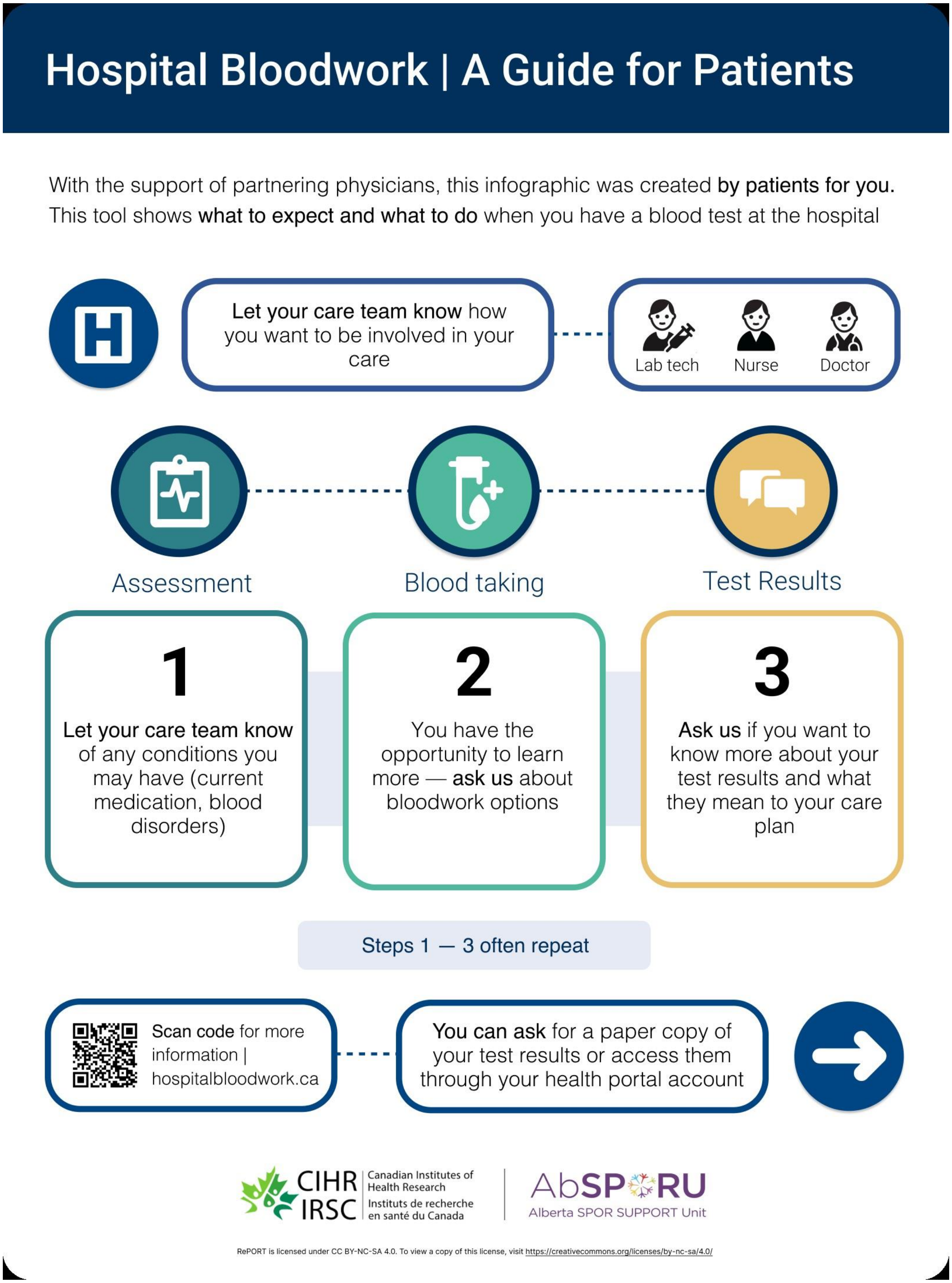

4b. Patient Video

www.hospitalbloodwork.ca/tools
