# Supplementary material for: Re-Purposing the Ordering of Routine Laboratory Tests in Hospitalized Medical Patients (RePORT): protocol for a multicenter stepped-wedge cluster randomised trial to evaluate the impact of a multicomponent intervention bundle to reduce laboratory test over-utilization
Source: Implement Sci. 2024 Jul 2;19:45. doi: 10.1186/s13012-024-01376-6 (PMC11221016; doi:10.1186/s13012-024-01376-6)
Supplement: Supplementary file 3 — Supplementary Material 3. [file 13012_2024_1376_MOESM3_ESM.docx]

Survey for RePORT: Project Champions

Project Overview **Project Overview**
Laboratory Test Overuse (LTO) occurs when tests are ordered repetitively, without due consideration of impact on clinical status. Repetitive inpatient lab testing often provides limited value for patient outcomes while increasing healthcare costs, patient discomfort, and unnecessary transfusions and prolonging hospitalizations. Thus, there is a need to introduce appropriate laboratory test utilization techniques within our hospitals. **RePORT**aims to address this need through a comprehensive, multi-disciplinary, and multi-faceted intervention bundle that includes audit and feedback reports, clinician education, clinical decision support tool, patient infographic, among other tools. Through the support of a patient advisory council and project champions, we aim to:
 a. Implement the LTO bundle for hospitalized medical inpatients in 30 hospitals across Alberta & British Columbia
b. Evaluate the impact of the LTO bundle on laboratory test utilization, costs, patient safety outcomes, and patient and healthcare.

**RePORT** is sponsored by the Canadian Institutes of Health Research, Alberta Health Services-Medicine Strategic Clinical Network, and supported by the University of Calgary, University of Alberta, and University of British Columbia. It has been approved by the University of Calgary Conjoint Health Research Ethics Board (REB17-1215) and the University of British Columbia Clinical Research Ethics Board (Ethics ID no. H22-03005).

Project champions such as yourselves play an essential role in helping to implement our project within local hospitals. Through this survey, we hope to understand your views on our project and its feasibility in your organization and assess its sustainability.

Q1. To begin this survey, please indicate the hospital site that you work at where RePORT is being implemented:

________________________________________________________________

Q2. Please indicate your role/ position within your organization:

- Hospitalist (1)
- GIM Physician (2)
- Nurse Practitioner (3)
- Quality Consultant (4)
- Patient Care Manager (5)
- Unit Manager (6)
- Other; Please indicate in the text box below: (7) __________________________________________________

Q3. The goal of the next few questions is to evaluate the organizational readiness for our project.  Please read each statement carefully and select the column that most accurately reflects your opinion of the statement. It is important that you comment on every statement.

|  | Strongly disagree (1) | Disagree (2) | Neither agree nor disagree (3) | Agree (4) | Strongly agree (5) |
| --- | --- | --- | --- | --- | --- |
| Using appropriate lab test utilization techniques can improve my efficiency (1) |  |  |  |  |  |
| Using appropriate lab test utilization techniques can improve patient outcomes (2) |  |  |  |  |  |
| The effort I contribute to reducing lab testing matches the benefit I receive (3) |  |  |  |  |  |
| I understand the need to implement appropriate lab test utilization projects (4) |  |  |  |  |  |
| I see myself as a champion/leader for appropriate lab test utilization projects in my organization (5) |  |  |  |  |  |
| Appropriate utilization of lab testing can enhance a team approach to care (6) |  |  |  |  |  |

Q4. Overall I think:

|  | Strongly disagree (1) | Disagree (2) | Neither agree nor disagree (3) | Agree (4) | Strongly agree (5) |
| --- | --- | --- | --- | --- | --- |
| my organization is committed to making appropriate lab test utilization projects successful (1) |  |  |  |  |  |
| my organization has experienced too much change over the past year (2) |  |  |  |  |  |
| my organization has the flexibility to reorganize resources to address changing utilization needs (7) |  |  |  |  |  |
| the project team has clearly communicated what was expected of me related to this project (3) |  |  |  |  |  |
| the project team has given me opportunities to provide input into the early phases of implementation and change (4) |  |  |  |  |  |
| my organization and project team will provide adequate resources for appropriate lab test utilization projects (6) |  |  |  |  |  |
| my organization and project team has access to experts who understand both lab test utilization and healthcare (8) |  |  |  |  |  |

Q5. Overall, I think addressing lab test utilization through a multimodal bundle in my organization:

|  | Strongly disagree (1) | Disagree (2) | Neither agree nor disagree (3) | Agree (4) | Strongly agree (5) |
| --- | --- | --- | --- | --- | --- |
| is a flexible approach as it allows for growth and change (1) |  |  |  |  |  |
| can be compatible with other technologies I am using (2) |  |  |  |  |  |
| can improve health system and patient outcomes (3) |  |  |  |  |  |

Q6. The goal of this part of the survey is to help us plan for sustainability of improvement efforts and recognize and understand key barriers for sustainability. At this stage, your input will enable us to identify and improve areas that requires strengthening right from the start. This survey is based on the NHS Sustainability Model and Guide. Please read each statement carefully and select the column that most accurately reflects your opinion of the statement. It is important that you comment on every statement.

|  | Strongly disagree (1) | Disagree (2) | Agree (3) | Strongly agree (4) | I don't know (5) |
| --- | --- | --- | --- | --- | --- |
| We can demonstrate that reducing the overuse of laboratory tests has a wide range of benefits beyond helping patients, for example by reducing waste and saving healthcare expenditures. (1) |  |  |  |  |  |
| Benefits of reducing the overuse of laboratory tests are widely communicated, immediately obvious, supported by evidence and believed by collaborators. (2) |  |  |  |  |  |
| Efforts to reduce the overuse of laboratory tests can link in and even support other organizational changes. It would not be disrupted if specific individuals or groups left the project. (3) |  |  |  |  |  |
| There is a system in place to provide evidence of impact, including benefits analysis, monitor progress and communicate the results. This is set up to continue beyond the formal life of the project. (4) |  |  |  |  |  |
| Healthcare providers have been involved from the beginning of the change process. They have helped to identify any skill gaps and have been able to access training and development so that they are confident and competent in the new way of working. (5) |  |  |  |  |  |
| Healthcare providers can share their ideas regularly. They believe that reduction of laboratory test overuse is a better way of doing things. (7) |  |  |  |  |  |
| Organizational leaders are highly involved and visible in their support of the change process. They use their influence to communicate the impact of the work and to break down any barriers. (8) |  |  |  |  |  |
| Clinical leaders are highly involved and visible in their support of the change process. They use their influence to communicate the impact of the work and to break down any barriers. (15) |  |  |  |  |  |
| The goals of the change are clear and have been shared widely. They are consistent with and support the organization’s strategic aims for improvement. This site has demonstrated successful sustainability of improvements before and has a ‘can do’ culture. (16) |  |  |  |  |  |
| Healthcare providers are confident and trained in the new way of working. Facilities and equipment are all appropriate to sustain the new process. (19) |  |  |  |  |  |
